# A TGFβR inhibitor represses keratin-7 expression in 3D cultures of human salivary gland progenitor cells

**DOI:** 10.1038/s41598-022-19253-x

**Published:** 2022-09-02

**Authors:** Eric W. Fowler, Emmett J. van Venrooy, Robert L. Witt, Xinqiao Jia

**Affiliations:** 1 Department of Materials Science and Engineering, University of Delaware, Newark, DE 19716 USA; 2 Department of Biological Sciences, University of Delaware, Newark, DE 19716 USA; 3 Helen F. Graham Cancer Center and Research Institute, Christiana Care, Newark, DE 19713 USA; 4 Department of Biomedical Engineering, University of Delaware, Newark, DE 19716 USA; 5 Delaware Biotechnology Institute, 590 Avenue 1743, Newark, DE 19713 USA

**Keywords:** Biotechnology, Cancer, Stem cells, Engineering, Materials science

## Abstract

Salivary gland tissue engineering offers an attractive alternative for the treatment of radiation-induced xerostomia. Key to the success of this approach is the maintenance and expansion of secretory acinar cells in vitro. However, recent studies revealed that in vitro culture of primary salivary gland epithelial cells led to undesirable upregulation of the expression of keratin-7 (K7), a marker of ductal phenotype and frequently associated with cellular stress. We have previously shown that hyaluronic acid (HA)-based, RGDSP-decorated hydrogels support the 3D growth and assembly of primary human salivary gland stem/progenitor cells (hS/PCs). Here, we investigate whether the RGDSP culture also promotes K7 expression, and if so, what factors govern the K7 expression. Compared to hS/PCs maintained in blank HA gels, those grown in RGDSP cultures expressed a significantly higher level of K7. In other tissues, various transforming growth factor-β (TGF-β) superfamily members are reported to regulate K7 expression. Similarly, our immunoblot array and ELISA experiments confirmed the increased expression of TGF-β1 and growth/differentiation factor-15 (GDF-15) in RGDSP cultures. However, 2D model studies show that only TGF-β1 is required to induce K7 expression in hS/PCs. Immunocytochemical analysis of the intracellular effectors of TGF-β signaling, SMAD 2/3, further confirmed the elevated TGF-β signaling in RGDSP cultures. To maximize the regenerative potential of h/SPCs, cultures were treated with a pharmacological inhibitor of TGF-β receptor, A83-01. Our results show that A83-01 treatment can repress K7 expression not only in 3D RGDSP cultures but also under 2D conditions with exogenous TGF-β1. Collectively, we provide a link between TGF-β signaling and K7 expression in hS/PC cultures and demonstrate the effectiveness of TGF-β inhibition to repress K7 expression while maintaining the ability of RGDSP-conjugated HA gels to facilitate the rapid development of amylase expressing spheroids. These findings represent an important step towards regenerating salivary function with a tissue-engineered salivary gland.

## Introduction

More than 600,000 head and neck cancer cases are diagnosed yearly, for which radiation is a common successful therapy^1,2^. Unfortunately, the salivary gland can be damaged in this process due to its proximity to the field of irradiation^1,3^. Salivary glands are composed of acinar cells that produce saliva and ductal cells that modify the saliva and direct its flow^1,4^. When acinar cells are damaged, they reduce or cease saliva production, resulting in salivary gland dysfunction that impedes one's ability to speak, swallow, masticate, and compromises oral health, ultimately impairing patients’ well-being^5,6^. Currently, approved therapies aim to increase saliva production from the remaining acinar cells; however, these methods do not offer regenerative stimuli to increase the acinar cell population^7,8^. There is a critical need for treatments that can restore salivary gland function and improve patients’ quality of life.

Tissue engineering offers regenerative opportunities for the treatment of salivary gland dysfunction; however, the isolation and expansion of saliva producing acinar cell cultures has remained challenging. In particular, in vitro culture of salivary gland cells has been reported to lead to loss of the secretory acinar characteristics and increased expression of markers characteristic of a ductal identity^9–15^. Recently, it was shown that stress or injury can stimulate the expression of ductal specific keratins, including keratin-7 (K7) and its transcript *KRT7*^9^. Abnormal keratin expression in injured, differentiated tissues is recognized as a stress response where keratin and other intermediate filaments increase survival by decreasing pro-apoptotic cell signaling^16–18^.

While factors governing K7 expression in the salivary gland have not been reported, transforming growth factor-beta (TGF-β) signaling has been implicated^1,19–21^, and it is well documented that TGF-β superfamily members regulate K7 expression in other tissue contexts^22–28^. TGF-β1 can induce cellular stress by inducing epithelial-to-mesenchymal transition (EMT)^29–31^ or by promoting cell cycle arrest^32,33^. During EMT, epithelial cells de/transdifferentiate and take on the characteristics of mesenchymal cells. Canonical TGF-β signaling is carried out through the phosphorylation and nuclear import of SMAD 2 and SMAD 3 (SMAD 2/3)^30,31,34^, and in ovarian cancer cells, a TGF-β1-SMAD 2/3-K7 mechanism has been described^26^. In non-malignant epithelial cells, TGF-β1 can induce senescence with irreversible cell cycle arrest. Cells undergoing senescence often secrete high levels of inflammatory cytokines, including a divergent member of the TGF-β superfamily, growth/differentiation factor-15 (GDF-15, *GDF15*), in a state termed the senescence-associated secretory phenotype (SASP)^32,35–37^. Elevated expression of TGF-β1 and GDF-15 have been reported in irradiated salivary glands^38,39^, where senescence is known to play a major role in disrupting salivary gland regeneration^29,40,41^. SMAD 2/3 signaling can also be influenced by the local ECM microenvironment by potentiating interactions with the mechanically activated Yes Associated Protein (YAP)^42–44^. YAP promotes TGF-β signaling by enhancing the nuclear retention of SMAD 2/3^42,44^.

We have isolated human salivary stem progenitor cells (hS/PCs) from parotid gland biopsies and confirmed their stem/progenitor phenotype^45^. We have also developed hyaluronic acid (HA)-based hydrogels that support the 3D culture of hS/PCs under defined conditions and identified a mechanical stiffness regime that promotes the development of multicellular spheroids^46^. Recently, we demonstrated that HA matrices with a covalently immobilized RGDSP peptide, a fibronectin-derived integrin-binding ligand, promoted the rapid 3D expansion of amylase-expressing hS/PCs^47^. However, the expression of K7 as the 3D cultures expand remains unexplored.

To validate our tissue engineering approach, we systematically analyzed the expression of K7 and the associated cellular stress in our 3D cultures. We found that RGDSP modified hydrogels increased K7 expression compared to the control peptide-free HA hydrogels. To mitigate K7 expression, we investigated its potential upstream mediators. Using a TGF-β superfamily member array, we discovered elevated TGF-β1 expression in RGDSP cultures, which was corroborated with increased nuclear SMAD 2/3 levels. We found that while SMAD 2/3 accumulated in the nucleus, YAP remained cytoplastic in RGDSP cultures. Using a model 2D system, we confirmed that exogenous TGF-β1 promoted K7 expression as nuclear SMAD 2/3 accumulated, both of which were suppressed when the medium was supplemented with a TGFβR inhibitor (A83-01). Finally, we demonstrated that K7 expression arose under conditions of cellular stress rather than an indicator of increased ductal phenotype under these contexts. This work shows that during in vitro culture of hS/PCs, TGF-β1 can induce K7 expression, and medium supplementation with A83-01 can be used to repress K7 expression.

## Results

### RGDSP cultures developed a mixed phenotype with increased K7 and α-amylase expression

**Figure 1:**
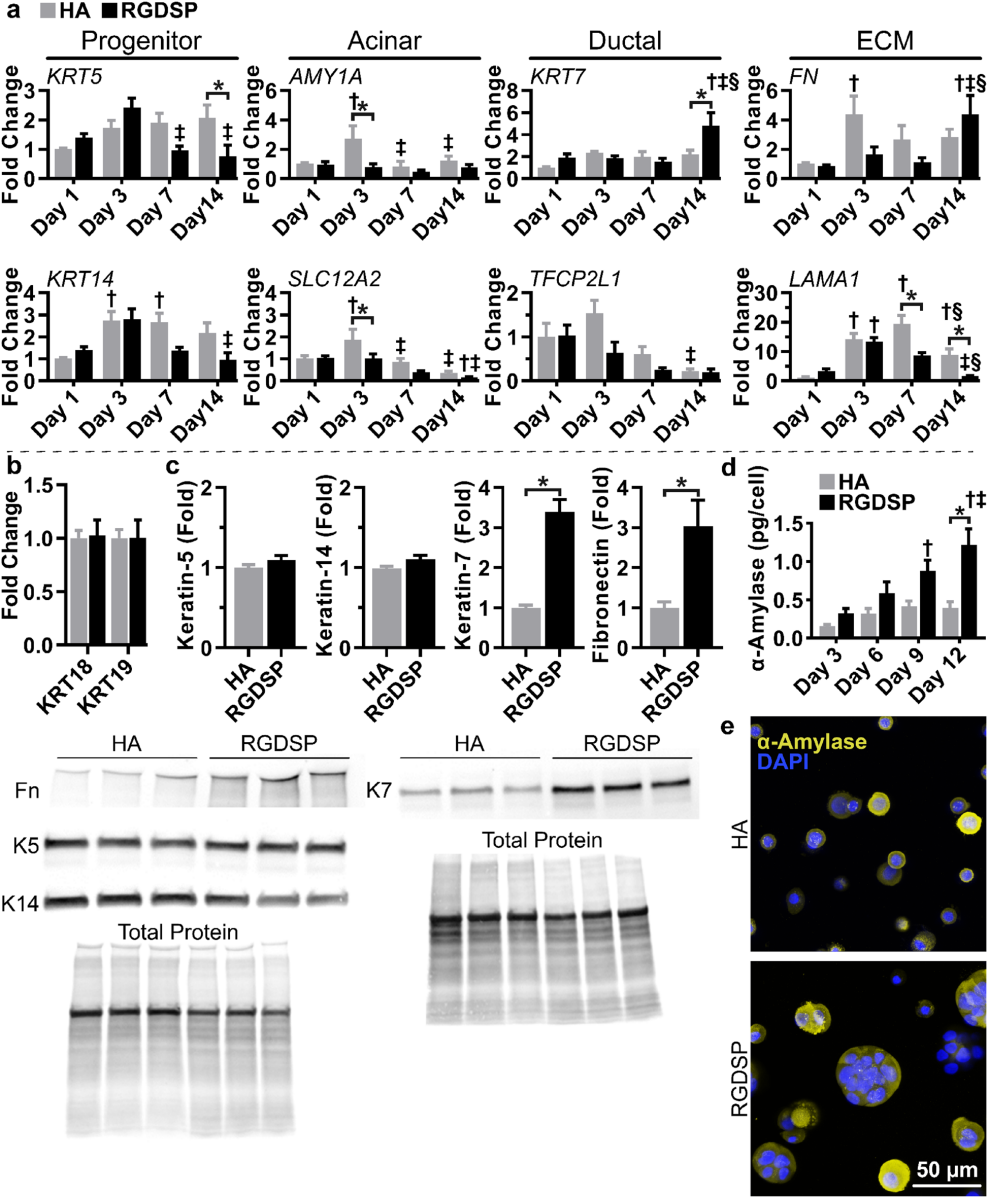
RGDSP gels promote the development of multicellular spheroids with increased K7 and α-amylase expression. (**a**) Temporal gene expression profile via qPCR analysis of *KRT5*, *KRT14*, *SLC12A2*, *KRT7*, *TFCP2L1*, *FN*, and *LAMA1*. (**b**) Expression of *KRT18* and *KRT19* on day 14 as analyzed by qPCR. (**c**) Western blot analysis for K5, K14, K7, and fibronectin on day 14. Blotting for fibronectin, K5, and K14 was performed sequentially on the same membrane (Fig. S7a–d). Blotting for K7 was performed separately (Fig. S7e,f). Triplicate measurements are presented from technical replicates extracted from different hydrogels. (**d**) Secreted α-amylase quantified by ELISA. (**e**) Immunocytochemistry (ICC) detailing uniform expression of α-amylase in day 14 spheroids. Single channel images are presented in Fig. S8. Quantification was conducted from 3 independent experiments, each with 3 technical repeats. Error bars represent SEM. Two-way ANOVA was conducted on data presented in (**a**) and (**c**), followed by Tukey’s multiple comparisons test. *p < 0.05 between HA and RGDSP cultures at the same time points. ^†,‡,§^*p* < 0.05 from days 1, 3, and 7 measurements of the same data set, respectively for (**a**) and days 3, 6, 9 for (**c**). Two-tailed Student’s t-tests were conducted on data appearing in (**b**, **c**), *p < 0.05.

Hydrogel precursors, including thiolated (HA-SH) and acrylated HA (HA-AES), as well as maleimide-functionalized RGDSP peptide (MI-RGDSP), were prepared as previously reported^47^. Cellular constructs were produced by dispersing hS/PCs in a solution containing HA-SH or HA-SH/MI-RGDSP before adding the HA-AES solution, resulting in HA or RGDSP cultures, respectively. To assess if the 3D hydrogel cultures promoted a K7-pro-ductal phenotype or reduced characteristics of an acinar phenotype, we characterized the temporal gene expression dynamics of salivary gland differentiation markers^47^ with qPCR (Fig. 1a). Keratins expressed by salivary gland progenitors, keratin-5 (K5, K*RT5*) and keratin-14 (K14, *KRT14*)^6,48,49^, presented similar temporal profiles that were unique to each hydrogel condition. There was a moderate increase in *KRT5*/*KRT14* expression in HA cultures on day 1 that was sustained to day 14. *KRT5*/*KRT14* expression in RGDSP cultures similarly increased on day 3; however, its expression decreased thereafter, with *KRT5* expression levels decreasing significantly below that observed in HA cultures. Analysis of the gene expression profile of acinar markers, α-amylase (*AMY1A*) and the Na-K-Cl ion transporter (*SLC12A2*)^1,6,49^, revealed different trends. *AMY1A* expression was relatively stable over time in both HA and RGDSP cultures. Although *AMY1A* expression was elevated on day 3 in HA cultures, it returned to the basal level on day 7. HA cultures maintained *SLC12A2* expression through day 7; however, on day 14, *SLC12A2* was downregulated with respect to its expression level on day 1. Comparatively, loss of *SLC12A2* expression occurred earlier in RGDSP cultures, with a significant reduction in expression occurring on day 7, and a further decrease on day 14.

Next, we analyzed the temporal expression of *KRT7* and a transcription factor involved in duct regulation, *TFCP2L1*^1,6,50^. HA and RGDSP cultures stably expressed *KRT7* until day 14, where RGDSP promoted a 2.2-fold higher *KRT7* expression relative to the day-14 HA cultures (4.8-fold increase when normalized to the expression level of HA cultures on day 1). However, a corresponding increase in the expression of the K7 dimerization partners, keratin-18 (*KRT18*) and keratin-19 (*KRT19*)^51,52^, was not detected in RGDSP cultures on day 14 (Fig. 1b). *TFCP2L1* expression was maintained in HA cultures until day 14, when it was downregulated relative to the normalized values of HA cultures on day 1. The expression of *TFCP2L1* was maintained across the culture time, and significant variations in expression between HA and RGDSP cultures were not observed.

Finally, the gene expression dynamics of fibronectin (*FN*), required in salivary gland development^53^, and laminin, alpha-1 subunit (*LAMA1*), which is associated with the maintenance of the acinar phenotype^54,55^, was also analyzed. *FN* and *LAMA1* were upregulated at days 3–7 under both culture conditions relative to the expression level by HA cultures on day 1. However, by day 14, *FN* was upregulated, and *LAMA1* was downregulated in RGDSP cultures.

Protein level confirmation of these findings was made by western blot analyses of K5, K14, K7, and fibronectin (Fig. 1c). Although *KRT5* mRNA expression was significantly lower in RGDSP cultures on day 14, we did not detect a loss in K5 expression on day 14 at the protein level. Furthermore, the expression of K14, the predominant heterodimeric partner of K5^51,52^, was also retained in the RGDSP cultures at day 14. However, K7 and fibronectin levels were increased by ~ threefold (*p* < 0.05) in RGDSP cultures, in agreement with the corresponding mRNA expression profiles. As increased ductal K7 expression might be associated with the loss of the secretory function, amylase expression was evaluated via ELISA. RGDSP cultures produced approximately twofold more amylase per cell on day 12 than HA cultures (Fig. 1d). However, the immunostaining pattern of α-amylase indicated that α-amylase was uniformly expressed throughout both HA and RGDSP cultures and was not restricted to a specific cell population (Fig. 1e). Similarly, K7 was also uniformly expressed in RGDSP cultures (Fig. S2). Collectively, these findings indicate that RGDSP increased secretory amylase secretion while also enhancing expression of the K7.

### TGF-β1 expression and nuclear SMAD 2/3 localization correspond to increased K7 expression

After confirming the development of the K7+ phenotype in RGDSP cultures, we set out to identify potential upstream regulators of K7. Literature mining indicates that members of the TGF-β family are responsible for activating K7 expression^22–28,56^. We used an immunoblot array to evaluate the production of TGF-β superfamily members in the medium collected from HA and RGDSP cultures on days 6 and day 14 (Fig. S3c–e). TGF-β1, GDF-15, and transforming growth factor-beta-induced protein (TGFBI/BIGH3) were the most highly detected TGF-β superfamily members in both HA and RGDSP cultures at both time points (Fig. S3e). TGF-β1 and GDF-15 were the only TGF-β superfamily members that increased expression from day 6 to day 14 in RGDSP cultures. Furthermore, only TGF-β1 was produced at higher levels in RGDSP cultures, relative to HA cultures, on day 14 when K7 expression was highest.

**Figure 2:**
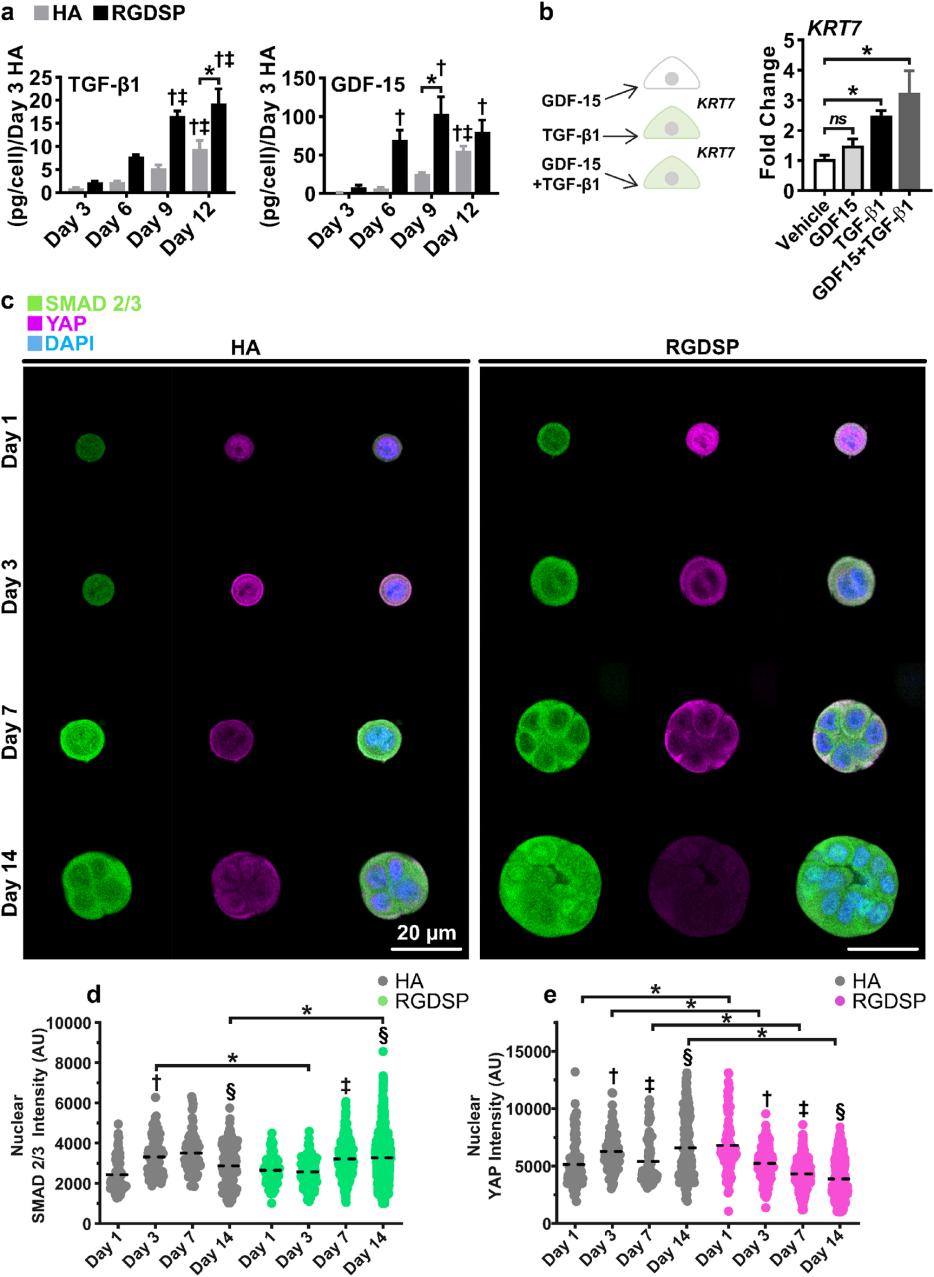
RGDSP cultures expressed high levels of TGF-β1 and GDF-15 that correlate with increased nuclear SMAD 2/3. Only TGF-β1 is required for *KRT7* expression in 2D cultures. (**a**) ELISA experiments were conducted targeting soluble TGF-β1 and GDF-15 in cell culture medium after 3, 6, 9, and 12 days of culture (n = 9, 3 independent experiments, each with 3 technical repeats). (**b**) hS/PCs were cultured with TGF-β1, GDF-15, and TGF-β1 + GDF-15 for 48 h on 2D substrates, and the expression of K7 was resolved with qPCR. Data averaged from 3 independent experiments, each with 3 technical repeats. Significance determined from one-way ANOVA followed by a Dunnett’s test, * indicates *p* < 0.05 relative to vehicle control. (**c**) Fluorescent microscopy images detailing hS/PC nuclear YAP, SMAD 2/3 and nuclei on days 1, 3, 7, and 14. Volumetric fluorescent microscopy was conducted, and representative single plane images are presented. Single channel images are presented in Fig. S9. (**d**,**e**) 3D rendering was performed with Imaris 3D-4D software for the quantification of nuclear SMAD 2/3 (**d**) and YAP (**e**) signals. Filled circles represent individual nuclei, and the dashed black line indicates the mean value of each data set. Error bars represent SEM in all cases. Number of images analyzed for HA cultures: 103 (day 1), 107 (day 3), 85 (day 7) and 225 (day 14); Number of images analyzed for RGDSP cultures: 104 (day 1), 150 (day 3), 428 (day 7) and 1534 (day 14). Analysis was conducted from 3 independent experiments. Two-way ANOVA was performed on data presented in (**a**) and (**d**, **e**) followed by Tukey’s multiple comparisons test. * indicates *p* < 0.05 between HA and RGDSP at the same time points. For (**a**), ^†,‡  ^*p* < 0.05 from day 3, and day 6 measurements of the same data set, respectively. In (**d**, **e**), ^†,‡,§ ^indicates *p* < 0.05 from day 1, 3, and day 7 measurements of the same data set, respectively.

We then conducted TGF-β1 and GDF-15 ELISA to confirm the array findings and assess the temporal cytokine expression dynamics (Fig. 2a). On days 3–12, HA and RGDSP cultures increased TGF-β1 expression by 9 and 19 folds, respectively. However, RGDSP cultures maintained a higher level of TGF-β1 expression relative to HA cultures (> 2 folds) throughout the entire culture period. Notably, a 7- and a 25-fold increase in GDF-15 expression, relative to the initial day 3 HA levels, was observed at days 6 and 9 in HA cultures. However, RGDSP cultures increased 70- to 103-fold over the same period. Interestingly, GDF-15 levels continued to rise in HA cultures, and by day 12 were no longer expressed at significantly different levels in HA and RGDSP cultures.

Since TGF-β1, GDF-15, and K7 were highly expressed in RGDSP cultures, we queried whether exogenous TGF-β1 (10 ng mL^**−**1^)^57,58^ or GDF-15 (100 ng mL^**−**1^)^59,60^ could induce *KRT7* expression. hS/PCs were maintained on 2D substrates with each cytokine independently and in combination for 48 h before qPCR analyses was conducted (Fig. 2b). Treatment with GDF-15 alone did not alter *KRT7* expression at the transcript level. In contrast, TGF-β1 induced a significant increase of *KRT7* expression that was sustained in the presence of GDF-15 (GDF-15 + TGF-β1). This result suggests that TGF-β1 is capable of stimulating *KRT7* expression.

To further rule out the possibility of GDF-15 in promoting K7 expression, we next inspected our cultures for signs of SASP, which is directly correlated to high levels of GDF-15^36^, and is associated with decreased salivary gland function^38,40,61^. Specifically, we compared the expression of a panel of SASP genes [*IL6*^36,40,41,61,62^*,* (*CXCL8*, IL8)^35,61,62^, *IL10*^63^, *IL1B*^62^*,* (*CDNK1A,* P21)^40,41^, (*CDNK2A*, P16)^40^, (*TP53,* P53)^32,35^, *GDF15*^36,61^*,* *SERPINE1*^35,36,41^*,* and *MMP1*^36,37^] in HA and RGDSP cultures on day 14 to 2D cultures stimulated by TGF-β1, GDF-15, and TGF-β1 + GDF-15 (Fig. S4a,b). The expression of senescent and inflammatory factors was largely governed by TGF-β1 in 2D cultures, while the activity of GDF-15 was limited to increasing the expression of *IL1B*. With regard to the cell cycle inhibitors that are indicative of senescence^35–37,40,41^, TGF-β1 stimulation significantly increased the expression of cyclin-dependent kinase inhibitor 2A (*CDKN2A)* and tumor protein P53 (*TP53*) in 2D cultures. However, RGDSP cultures did not exhibit elevated levels of *CDKN2A* or *TP53*, suggesting growth arrest had not occurred. TGF-β1 significantly enhanced *IL6* expression in 2D cultures; however, RGDSP cultures promoted increased *CXCL8* expression without stimulating *IL6* expression. These results suggest that interleukins are not responsible for *KRT7* expression. Moreover, elevated expression of *SERPINE1*, a TGF-β1 target gene^25,30^, was observed in RGDSP cultures as well as TGF-β1 stimulated 2D cultures, further confirming that TGF-β1, not GDF-15 or SASP, was the dominant factor governing emergence of the K7+ phenotype.

We hypothesize that integrin-mediated adhesion via RGDSP can enhance mechanotransduction, by nuclear localization of YAP, to promote TGF-β signaling in RGDSP cultures^42^. To this end, we characterized TGF-β signaling and examined the role of mechanotransduction in inducing K7 expression by targeting SMAD 2/3 and YAP with double immunofluorescence (Fig. 2c). Nuclear localization of YAP or SMAD 2/3 is indicative of increased YAP and TGF-β signaling, respectively^44,64,65^. In HA cultures, nuclear SMAD 2/3 signal was increased at day 3, but returned to the baseline level by day 14 (Fig. 2d). Under the same condition, elevated nuclear YAP was detected on days 3 and 14. In RGDSP cultures, nuclear YAP intensity peaked on day 1, but continuously decreased thereafter (Fig. 2e).

A similar trend was observed for cytoplasmic YAP (Fig. S5a). As nuclear YAP decreased in RGDSP cultures, nuclear SMAD 2/3 increased significantly at day 7 and furthermore at day 14, resulting in significantly higher levels at day 14 than observed in HA cultures. However, cytoplasmic SMAD 2/3 levels did not significantly increase in RGDSP cultures (Fig. S5b). These findings confirm that the increased TGF-β signaling in RGDSP cultures corresponds to the rising TGF-β1 levels and increased K7 expression. Conversely, RGDSP cultures do not sustain elevated YAP expression to drive TGF-β signaling and K7 expression.

To confirm the microscopy findings, we further compared the temporal nuclear localization of SMAD 2/3 and YAP with other TGF-β and YAP targets (Fig. S5c). Irrespective of nuclear SMAD 2/3 and YAP expression, HA and RGDSP cultures were defined by an initial *ADAM10*/*ADAM17* activation with the expression of EGFR ligands (*EREG, AREG, HBEGF, TGFA*), followed by a transition to *MMP1* expression as the culture time proceeded*.* In HA cultures, the appearance of nuclear SMAD 2/3 and YAP together at day 3 dominated the transcriptional events where *KRT7*, *FN*, and *ITGAV* were maximally expressed. However, in RGDSP cultures, nuclear SMAD 2/3 expression was highest on day 14, corresponding to the highest *KRT7*, *FN,* and *ITGAV* expression. In RGDSP cultures, nuclear YAP expression clearly proceeded *YAP1* and *CTGF* expression; however, in HA cultures, the presence of both SMAD 2/3 and YAP in the nucleus at day 3 maximized *YAP1* and *CTGF* expression. Collectively, the temporal expression of SMAD 2/3 and YAP was confirmed both at the transcript and protein levels.

### Loss of nuclear YAP stimulates GDF15 expression and downregulates TGF-β target genes

**Figure 3:**
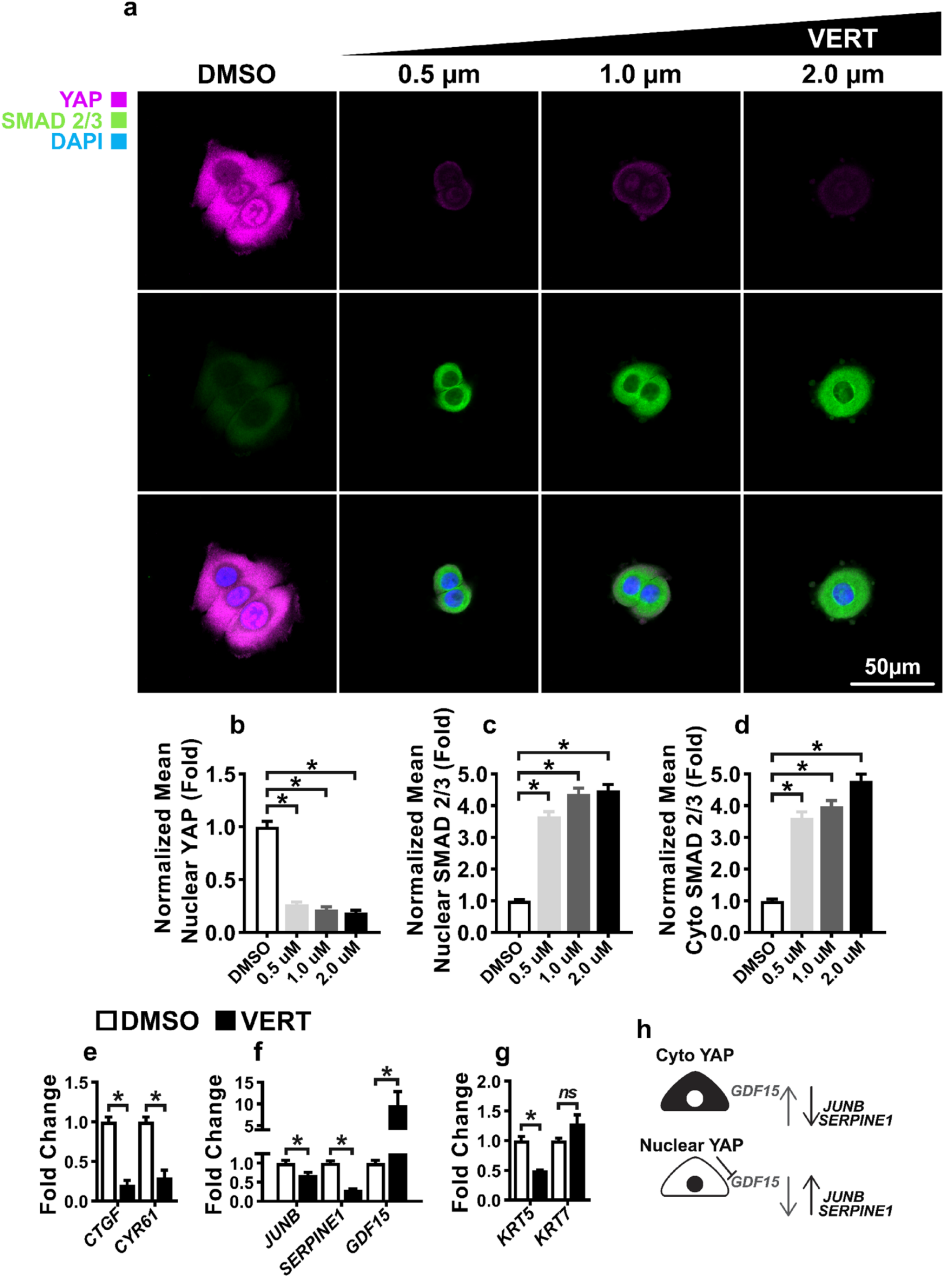
Loss of nuclear YAP stimulates *GDF15* expression and downregulates TGF-β1 target genes. (**a**) hS/PCs were cultured with verteporfin (VERT, 0.5, 1.0, and 2.0 µM) and the DMSO vehicle control for 24 h before SMAD 2/3 and YAP were visualized by ICC. Nuclei were counterstained with DAPI. Single channel images are presented in Fig. S10. (**b**–**d**) Image quantification was performed with ImageJ to resolve nuclear YAP (**b**), nuclear SMAD 2/3 (**c**), and cytoplasmic SMAD 2/3 (**d**). Number of images analyzed: n_DMSO_ = 33, n_0.5_ = 24, n_1.0_ = 19, n_2.0_ = 17, from 3 independent experiments, significance determined from one-way ANOVA followed by a Dunnett’s test, * indicates *p* < 0.05 relative to DMSO control. (**e**–**g**) hS/PCs were treated with 1 μM VERT for 24 h and mRNA expression of YAP target genes: *CTGF*, *CYR61* (**e**); TGF-β targets genes: *JUNB*, *SERPINE1*, and *GDF15* (**f**); and keratins: *KRT5*, *KRT7* (**g**) were resolved with qPCR (n = 9, 3 independent experiments, each with 3 technical repeats). (**h**) Schematic depiction detailing the role of nuclear YAP (black circle) in supporting TGF-β signaling (*JUNB*, *SERPINE1*) and repressing *GDF15* expression. Error bars represent SEM in all cases. Two-tailed Student’s t-tests were conducted on data appearing in (**e**–**g**). * indicates *p* < 0.05 in all cases.

We have shown that TGF-β1 can stimulate *KRT7* expression in hS/PCs (Fig. 2b), and we also know that YAP is involved in maintaining the stem/progenitor status of various tissues^66,67^. Thus, we questioned if loss of nuclear YAP expression, as observed in RGDSP cultures, could be sufficient to induce the expression of K7^66,67^. To this end, a series of YAP inhibition studies were performed to investigate YAP-dependent TGF-β signaling. hS/PCs were maintained on 2D substrates with a verteporfin (VERT), an inhibitor of YAP-TEAD transcription^68,69^, at concentrations of 0.5, 1.0, and 2.0 µM for 24 h, in the absence of light, and the expression of nuclear SMAD 2/3 and YAP was resolved with fluorescent microscopy (Fig. 3a). As shown in Fig. 3b, VERT treatment effectively suppressed nuclear YAP expression. Compared to the vehicle controls, VERT at 0.5 μM decreased nuclear YAP expression by ~ 0.75 fold, with further reductions in YAP observed at 1.0 and 2.0 µM. Meanwhile, VERT treatment led to a significant increase in nuclear SMAD 2/3, with ~ 3.5 fold increase in response to 0.5 µM VERT (Fig. 3c). Of note, VERT treatment also increased the cytoplasmic SMAD 2/3 levels (Fig. 3d).

Next, qPCR was performed targetting TGF-β and YAP pathway transcripts to characterize the effects of YAP inhibition on TGF-β signaling in cultures receiving 1.0 µM of VERT (Fig. 3e–g). As expected, *CTGF* and *CYR61*, canonical YAP target genes^70^, were downregulated, confirming the repression of YAP signaling with VERT treatment (Fig. 3e). The TGF-β targets, *JUNB* and *SERPINE1,* were upregulated in hS/PC cultures after treatment with TGF-β1 (Figs. S4b, S6c), indicating these genes responded to TGF-β signaling in hS/PC cultures. YAP inhibition downregulated *JUNB* and *SERPINE1,* indicating the supporting role of YAP in the expression of these transcripts (Fig. 3h). *GDF15* was modestly upregulated (1.5-fold, *p* = *0.08*) in TGF-β1 treated cultures (Fig. S4b); however, it was increased by ~ ninefold in response to VERT-mediated YAP inhibition. Additionally, the role of YAP in the regulation of keratin expression was investigated (Fig. 3g). *KRT5* was downregulated with the loss of YAP, but alterations in *KRT7* expression was not detected. These findings indicate that YAP represses *GDF15* expression while supporting the expression of the TGF-β signaling components *JUNB* and *SERPINE1* (Fig. 3h).

### TGFβR inhibition represses TGF-β1 induced K7 expression in 2D cultures

**Figure 4:**
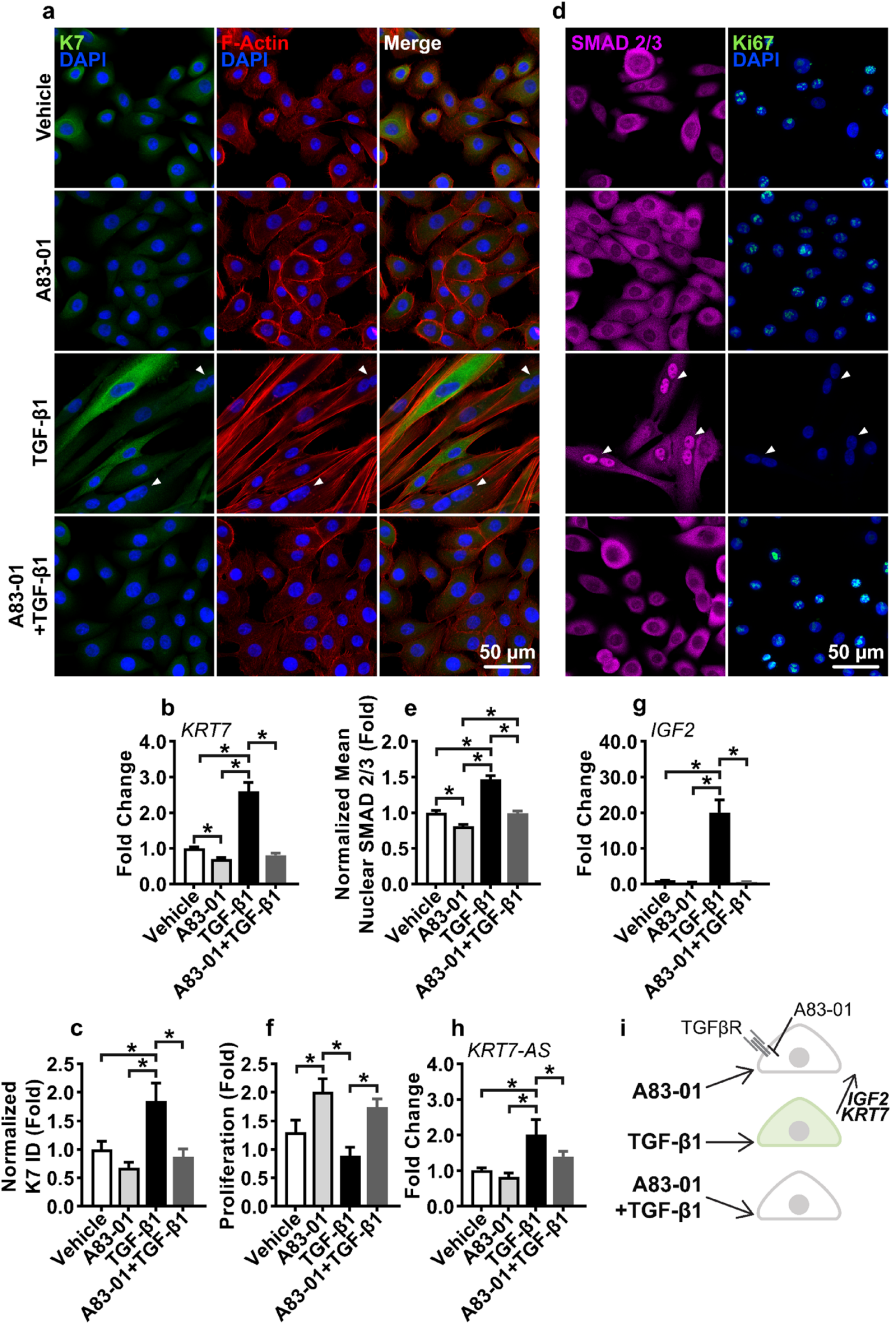
TGFβR inhibition represses TGF-β1 induced K7 expression. (**a**) hS/PCs were cultured with TGF-β1 and A83-01 for 48 h, and the expression of K7 and F-Actin was visualized with fluorescent microscopy. (**b**,**c**) A83-01 repressed TGF-β1 induced *KRT7* expression at the mRNA level (b, n = 9, 3 independent experiments, each with 3 technical repeats), and at the protein level as determined by image analysis conducted with ImageJ (c, ID: integrated density; number of images analyzed: n_Vehicle_ = 55, n_A83-01_ = 55, n_TGF-β1_ = 40, n_A83-01+TGF-β1_ = 45 from 3 independent experiments). (**d**) hS/PCs were cultured with TGF-β1 and A83-01 for 48 h, and SMAD 2/3 and Ki-67 expression were investigated with ICC. White arrows indicate binuclear cells in TGF-β1 treated cultures. Single channel images of (**a**) and (**d**) panels are presented in Figs. S11 and 12, respectively. (**e**) ImageJ-derived analysis indicated TGF-β1 stimulated nuclear SMAD 2/3 that was inhibited by A83-01. Number of images analyzed: n_Vehicle_ = 70, n_A83-01_ = 57, n_TGF-β1_ = 41, n_A83-01+TGF-β1_ = 56, from 3 independent experiments. (**f**) Proliferation was assessed by enumeration of DAPI stained nuclei in TGF-β1, and A83-01 treated cultures after 48 h of culture. At 24 h, n_Vehicle_ = 629, n_A83-01_ = 637, n_TGF-β1_ = 308, n_A83-01+ TGF-β1_ = 705 from 3 independent experiments. (**g**,**h**) hS/PC expression of *IGF2* (**g**) and *KRT7-AS* (**h**) with TGF-β1 and A83-01 treatment were investigated with qPCR. n = 9 from 3 independent experiments. Error bars represent SEM in all cases One-way ANOVAs were performed on data presented in (**b**), (**c**), and (**e**–**h**) followed by Tukey’s multiple comparisons test. * indicates *p* < 0.05 in all cases. (**i**) Schematic depiction of A83-01 inhibition of TGF-β1 stimulated K7 expression.

To ascertain whether decreasing TGF-β signaling could suppress K7 expression, model 2D studies were conducted with a TGFβR inhibitor, A83-01. hS/PCs were cultured for 48 h with TGF-β1 and 2 µM of A83-01, a concentration reported not to exhibit off target signaling inhibition^71^. As shown in Fig. 4a, TGF-β1-treated cells were strikingly larger and extended with fewer cell–cell contacts, taking on a mesenchymal spindle shape morphology. A83-01 inhibition promoted an epithelial phenotype with F-actin localized to cell–cell contacts. When provided in combination with TGF-β1, A83-01 prevented the TGF-β1-induced morphology changes. Immunofluorescence microscopy detailed a granular expression pattern of K7, which increased in response to TGF-β1 (Fig. 4b). Furthermore, A83-01 was sufficient to resist the TGF-β1 mediated increase in K7 expression. Similarly at the mRNA level, A83-01 inhibited the TGF-β1 stimulated *KRT7* expression while simultaneously decreasing *KRT7* expression below that of the vehicle control (Fig. 4c). Immunofluorescence microscopy was performed to demonstrate further that *KRT7*/K7 was correlated with the ability of TGF-β1 to induce nuclear SMAD 2/3, and inhibition of *KRT7*/K7 expression via A83-01 was also accompanied by reduced nuclear SMAD 2/3 (Fig. 4d,e). Furthermore, we found that hS/PCs treated with exogenous GDF-15 did not exhibit increased nuclear SMAD 2/3 (Fig. S4c), further indicating aberrant TGF-β signaling promotes K7 expression.

TGF-β1 is a cytostatic factor towards epithelial cells^32,33^, and with TGFβR inhibition by A83-01, hS/PCs responded with rapid proliferation (Fig. 4f). In addition, competitively inhibited cultures receiving A83-01 retained higher proliferation than cultures receiving only TGF-β1 (A83-01 + TGF-β1 vs TGF-β1). The non-G_0_ state cell cycle marker, Ki-67^72^, was absent in TGF-β1 treated cultures, although the DMSO controls were stained positive (Fig. 4d). Higher expression of Ki-67 indicates that cells could proliferate, but the DMSO cultures did not proliferate to a significantly high degree than the TGF-β-treated ones over the 48-h time span of the experiment (A83-01 vs TGF-β1, Fig. 4f). On the other hand, nuclear Ki-67 was present in cultures treated with both A83-01 and TGF-β1, further demonstrating the ability of A83-01 to inhibit TGF-β signaling. Under the cytostatic effects of TGF-β1, cytokinesis failure can occur, producing binuclear cells^73^, which were present in TGF-β1 treated cultures (arrowheads, Fig. 4a,d). This is associated with genome instability, loss of imprinting (LOI), and led to the investigation of the expression of a long non-coding antisense (AS) RNA, *KRT7-AS*, reported to regulate *KRT7*/K7 expression^73–75^. We determined that in response to TGF-β1, *IGF2*, a paternally imprinted gene of insulin-like growth factor 2 (IGF-2)^50,76^, was upregulated ~ 20 fold, and concordantly, *KRT7-AS* expression was induced (Fig. 4g,h). Furthermore, TGF-β1 modestly upregulated insulin-like growth factor 1 receptor (IGF-1R, *IGF1R*), yet the expression of the non-imprinted insulin-like growth factor 1 (IGF-1^77^, *IGF1*) was unaltered (Fig. S6a,b). A83-01 medium supplementation prevented TGF-β1 induced *KRT7* and *IGF2* expression (Fig. 4i), indicating that A83-01 was sufficient to inhibit the activation of genes associated with genome instability.

### TGFβR inhibition represses RGDSP induced K7 expression

**Figure 5:**
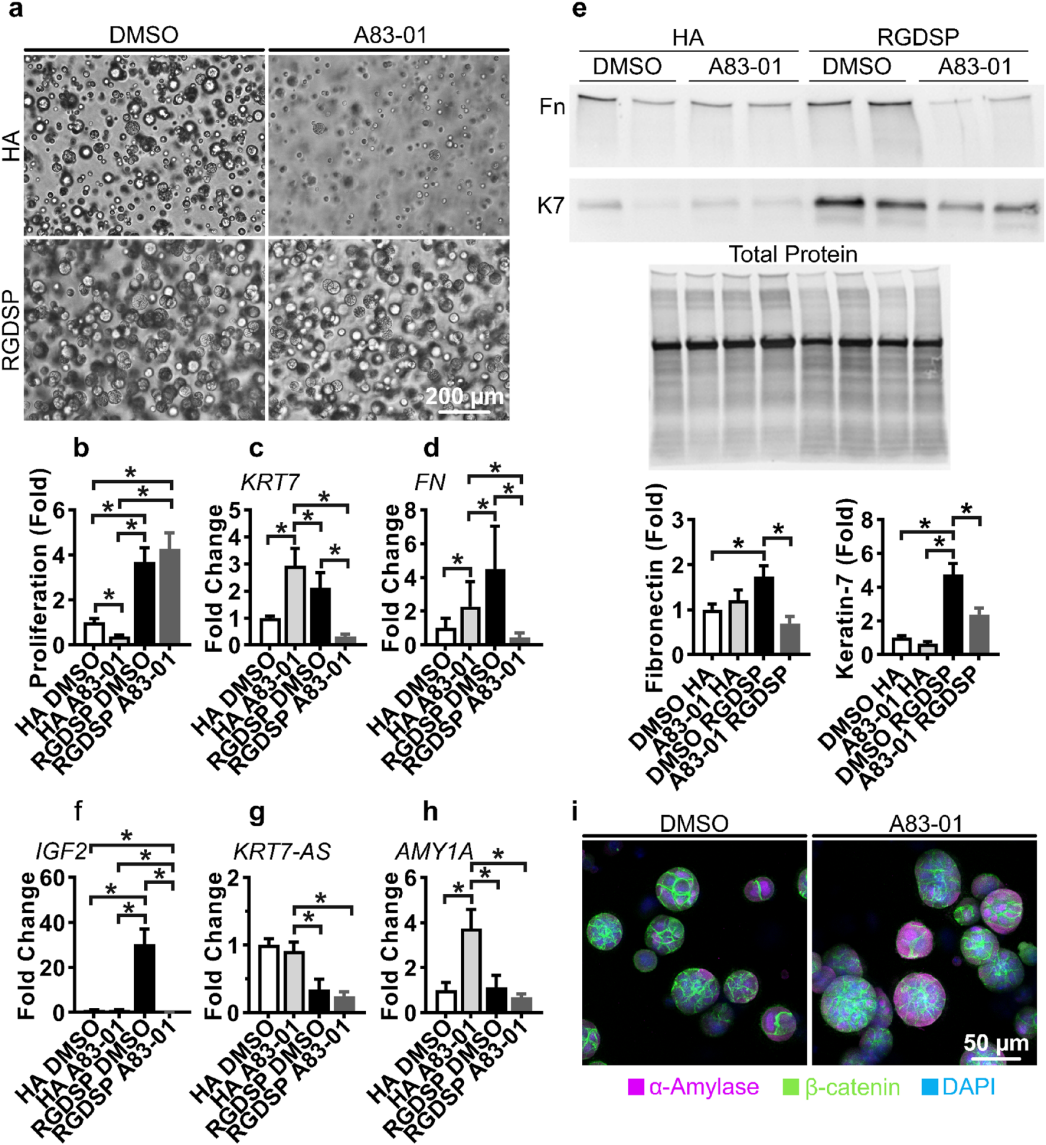
TGFβR inhibition represses RGDSP-induced K7 expression. (**a**) hS/PCs were cultured in HA and RGDSP hydrogels with A83-01 or the vehicle control (DMSO) for 14 days and visualized with bright field microscopy. (**b**) dsDNA, indicative of proliferation, was resolved from simultaneous TRIzol RNA/protein extractions using the Quant-iT PicoGreen assay. (**c**,**d**,**f**–**h**) hS/PCs were cultured in HA and RGDSP hydrogels for 14 days receiving A83-01 or DMSO, and expression of *KRT7* (**c**), *FN* (**d**), *IGF2* (**f**), *KRT7-AS* (**g**), and *AMY1A* (**h**) was assessed with qPCR. (**e**) hS/PCs were cultured in HA and RGDSP hydrogels for 14 days receiving A83-01 or DMSO, and western blotting was conducted to investigate K7 and fibronectin expression. Blotting for fibronectin and K7 was performed sequentially on the same membrane (Fig. S7g–i). Duplicate measurements are presented from technical replicates extracted from separate hydrogels. (**i**) hS/PCs were cultured for 14 days in RGDSP receiving A83-01 or DMSO, and fluorescent microscopy indicated that expression α-amylase and β-catenin was not suppressed by treatment with A83-01. Single channel images are presented in Fig. S13. Quantification was conducted from 3 independent experiments, each with 3 technical repeats. Error bars represent SEM in all cases. One-way ANOVAs were performed on data presented in (b-h) followed by Tukey’s multiple comparison test. * indicates *p* < 0.05 in all cases.

After associating nuclear SMAD 2/3 with increased *KRT7*/K7 expression and demonstrating that A83-01 can mitigate this response under 2D conditions, we investigated the effectiveness of A83-01 supplementation towards inhibiting TGF-β1 mediated K7 expression in RGDSP cultures. Spheroid formation was disrupted in A83-01-treated HA cultures (Fig. 5a). Proliferation, determined by DNA yield, in the HA + A83-01 cultures was reduced by ~ twofold, although a marginal increase in cell proliferation was observed in A83-01-treated RGDSP cultures (Fig. 5b). Although the proliferative properties of A83-01 were not maintained from 2 to 3D, the expression of *KRT7* and *FN* were repressed in A83-01 treated RGDSP cultures to the levels of the DMSO treated HA cultures (Fig. 5c,d).

Western blot analysis further confirmed that the RGDSP-promoted increase in K7 and fibronectin expression was repressible by inhibition of TGF-β signaling (Fig. 5e). Although A83-01 treated HA cultures exhibited increased *KRT7* and *FN* mRNA levels, increased protein levels of K7 and fibronectin were not detected (Fig. 5e).

On 2D substrates, we established the potential for TGF-β1 to induce *IGF2* and KRT7AS expression by hS/PCs. When profiling the entire culture period, a distinct temporal event was found to occur at day 14, when *IGF2* was upregulated ~ 30 fold in 3D RGDSP cultures (Fig. S6d). IGF2 expression also correlated (Pearson's r: 0.999, p = 0.005) with *KRT7* expression in RGDSP cultures. However, the expression of the *IGF1* or *IGF1R* genes did not follow the same trend (Fig. S6d–f). As expected, A83-01 suppressed the *IGF2* expression stimulated by RGDSP cultures, and *IGF2* expression was not activated by inhibiting TGF-β signaling in HA cultures either (Fig. 5f). However, the enhanced expression of the *KRT7-AS* transcript that was observed in response to TGF-β1 was not induced in the RGDSP cultures, suggesting this mechanism is not required for the observed increased *KRT7*/K7 expression brought on by RGDSP (Fig. 5g).

**Figure 6:**
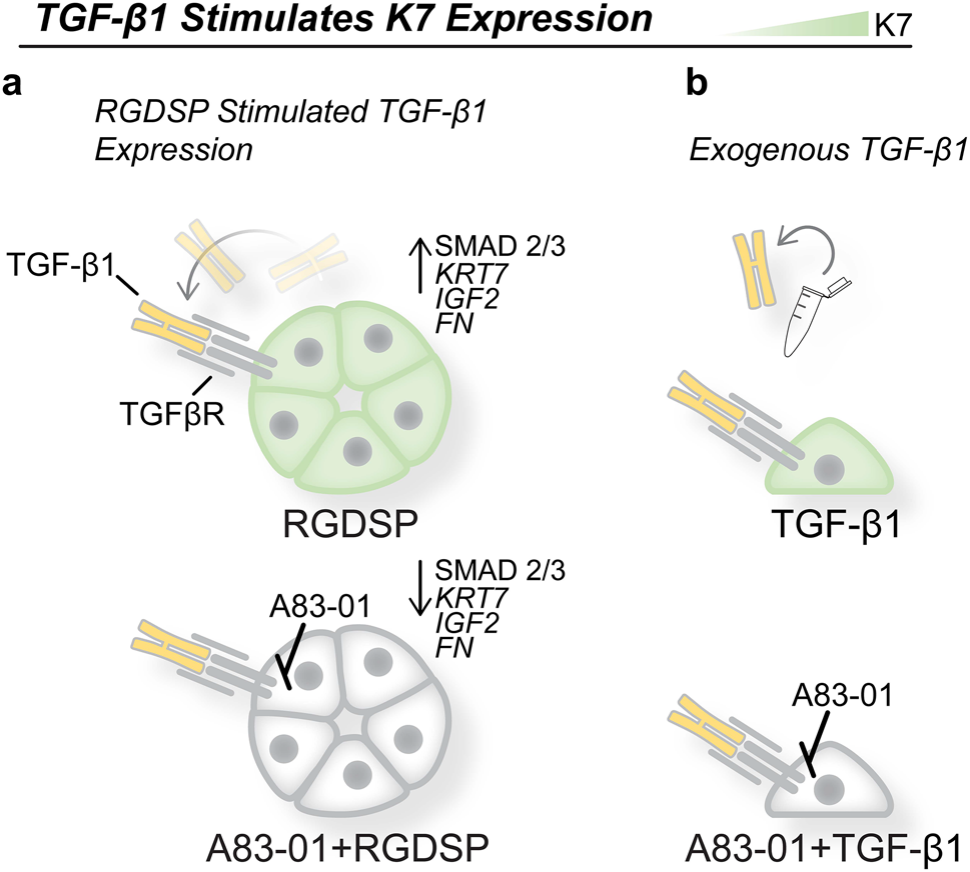
Schematic depiction of TGF-β1 stimulation of K7 expression in hS/PC cultures. (**a**) In 3D cultures, RGDSP stimulates TGF-β1 secretion to drive K7 expression, which is repressed by the addition of A83-01. (**b**) In 2D cultures, exogenous TGF-β1 stimulates K7 that is inhibited by the addition of A83-01.

qPCR and ICC results indicated TGF-β inhibition led to the repression of K7 and the maintenance of amylase expression (Fig. 5e–i). Furthermore, we found β-catenin remained localized to cell–cell junctions with TGF-β inhibition in RGDSP cultures (Figs. 5i, S13). Thus, we determined that the enhanced proliferation and amylase expression provided by RGDSP were maintained as K7 expression was suppressed with TGF-β signaling inhibition. Collectively we conclude that RGDSP stimulates increased TGF-β1 expression, which in turn induces K7 expression, analogous to the addition of exogenous TGF-β1 supplementation in 2D cultures (Fig. 6).

## Discussion

A preeminent obstacle for salivary gland tissue engineering during ex vivo expansion of primary salivary gland cultures is the difficulty in the maintenance of acinar identity; the decreased acinar identity is often accompanied with an increase in the expression of ductal associated keratin 7^9–15^. Previously we show that RGDSP-decorated HA gels promoted the rapid growth of amylase expressing cells, a pre-requisite for establishing an engineered gland using patient derived cells^47^. We report here that RGDSP peptide also promotes the expression of K7, which can be repressed by the TGFβR inhibitor, A83-01. We demonstrate that medium supplementation with A83-01 does not compromise the increased proliferation and amylase expression contributed by the RGDSP peptide. We discovered that increased K7 expression was accompanied by elevated TGF-β1 expression and nuclear SMAD 2/3 localization in RGDSP cultures. These observations were replicated in 2D cultures by treatment with exogenous TGF-β1. Based on the nuclear localization of SMAD 2/3, we further implicated its role in the expression of K7 by demonstrating that as A83-01 inhibited TGF-β1 mediated K7 expression, nuclear SMAD 2/3 levels were also suppressed. These findings indicate that during in vitro culture of primary salivary gland cells, K7 expression can be induced by conditions that stimulate TGF-β1 expression. We suggest that medium supplementation with A83-01 is a viable option to repress the expression of K7 in cultured salivary gland cells.

Media supplementation with TGF-β/SMAD inhibitors has found wide applicability in primary epithelial cell culture due to their ability to prevent epithelial differentiation and senescence^19,20,78,79^. Supplementing the medium of passage 9 murine submandibular gland cultures with a TGFβR inhibitor was found to increase aquaporin-5 (acinar marker) and amylase expression^19^. When murine submandibular gland cells were isolated and maintained with a TGFβR inhibitor, an enriched progenitor phenotype developed that exhibited decreased K7 and aquaporin-5 expression^20^. However, a TGF-β1-dependent induction of K7 expression in salivary gland cells has yet to be confirmed. In ovarian cancer cells, a similar TGF-β1-SMAD 2/3-K7 mechanism has been described^26^. Similarly, K7 is among stiffness responsive genes that can be induced by exogenous TGF-β2 in human endothelial cell cultures^27^. More broadly, TGF-β superfamily members regulate K7 expression across various tissues^22–28^. Thus, our findings are novel and supported by the known role of TGF-β signaling in the regulation of epithelial differentiation and K7 expression.

While K7 is a classical marker of a ductal phenotype in the native salivary gland, in the context of the current investigation, enhanced K7 expression does not necessarily suggest that hS/PCs in RGDSP-decorated HA gels progressed towards ductal cells. The transcript levels of acinar markers (*AMY1A* and *SLC12A2*) were not compromised in RGDSP cultures, nor did the ductal marker *TFCP2L1* increase as K7 expression increased. The RGDSP cultures did not lead to the development of a mixed acinar and ductal population either, as our ICC results show that amylase and K7 were homogeneously expressed in RGDSP cultures.

Overexpression of intermediate filaments, including keratin, desmin, nestin, and vimentin, is broadly recognized as a response to cellular stress^16,17^. The expression of intermediate filaments is reported to provide an anti-apoptotic signal as they act as a sponge for aberrant phosphorylation, provide scaffolding for 14-3-3 signaling regulatory proteins, and sequester apoptotic proteins^16,18^. Thus, there is strong evidence that K7 expression indicates a state of cellular stress. However, we cannot conclude that the TGF-β1 mediated K7 expression observed here indicates that RGDSP cultures are de/transdifferentiating to a ductal phenotype.

Prior reports have found increased expression of the mesenchymal marker vimentin with K7 during the isolation and culture of primary salivary gland cells^11,12^. Although TGF-β1 is a principal factor of EMT, we did not explore the role of EMT in our work and conclude that the TGF-β1-induced cellular stress is responsible for K7 expression. IGF2 is among transcripts that should not be expressed in healthy salivary glands and often found in salivary gland tumors^50,76^. We observed that *IGF2* was highly expressed in RGDSP cultures at the same time K7 expression was induced and treatment of 2D cultures with TGF-β1 replicated this outcome. Under both culture conditions, A83-01 inhibited the expression of *IGF2.* Collectively, these findings further suggest that TGF-β1 is responsible for the elevated *KRT7*/K7 and *IGF2* expression observed in RGDSP cultures. Additionally, the observation that *IGF2* and *KRT7* expression are stimulated together suggests that K7 expression is a response to cellular stress.

Our findings suggest that expression of GDF-15, an indicator of cellular stress including SASP^36,37,80^, can indicate conditions where K7 expression might be induced, yet exogenous GDF-15 did not activate *KRT7* expression in hS/PC cultures or promote nuclear localization of SMAD 2/3. SASP has been associated with reduced salivary gland function, and we found elevated expression of SASP markers in cultures expressing K7. However, the only elevated SASP marker present in both RGDSP cultures and TGF-β1 treated 2D cultures was *SERPINE1*. Given that *SERPINE1* is a known target of TGF-β1, these findings further suggest that TGF-β1 is responsible for the elevated K7 expression in RGDSP cultures. While K7 expression was observed alongside the expression of inflammatory markers, we could not confirm their requirement for the induction of K7 expression.

We found that RGDSP cultures promoted nuclear SMAD 2/3 accumulation as nuclear YAP was downregulated. The loss of nuclear YAP did correspond to increased GDF-15 expression in RGDSP cultures, and this was further demonstrated with VERT treatment. The finding that nuclear YAP inversely correlates with GDF-15 expression could indicate that dysregulated YAP expression is a response to mechanical cell stress. Our findings are supported by studies employing genetic overexpression, mechanical inactivation, and VERT inhibition, demonstrating the ability of YAP to suppress GDF-15^59,81,82^. A direct role for YAP in the regulation of K7 expression was not confirmed.

In this work, nuclear localization of SMAD proteins, rather than the phosphorylated forms, was used to correlate TGF-β signaling. Our attempt using phospho-SMAD 2/3 antibodies did not provide enough signal to robustly evaluate TGF-β signaling with fluorescence microscopy in 3D cultures. The antibody used here binds both phospho- and non-phosphorylated forms. Because phosphorylated SMAD 2/3 involved in active TGF-β1 signaling are localized in the nucleus, by restricting our analysis to the nucleus only, it is possible to indirectly evaluate TGF-β signaling using an antibody targeting the total proteins^83–85^. We will optimize experimental conditions in our future studies to enable direct analysis of phospho-SMAD 2/3.

Although we did not investigate how RGDSP promotes TGF-β1 expression, it is well known that TGF-β signaling is tightly linked to ECM composition. RGDSP can bind α_V_β3, and α_V_β5 integrins^86,87^, and α_V_ integrins are also known to activate TGF-β signaling^88–90^. Furthermore, increased protease expression and mechanotransduction can also promote TGF-β signaling^88,90^. From the biomaterials design perspective, the inflammatory properties of RGDSP can be mitigated by including its synergy sequence to target α_5_ as opposed to α_V_ integrin subunits^86,91–93^. Also, incorporating dual active and passive degradation mechanisms has successfully mitigated the inflammatory response arising from synthetic hydrogel culture^94^. Although imparting enzymatic degradation in synthetic hydrogel designs has been beneficial towards sustaining an acinar phenotype, it is only partially sufficient, and the underlying problem has not been identified^13,95^. Our findings will be realized in future hydrogel designs by incorporating properties to negate the signaling and overexpression of TGF-β1. Overall, our work suggests that balanced SMAD signaling is required to sustain salivary gland cultures, in agreement with prior findings^34,39^. We expect incorporating other known pro-acinar factors, FGF2, FGF7, FGF10, laminin-111, and Y-27632, will be further beneficial towards suppressing K7 expression and maintenance of the acinar phenotype^13,54,55^.

## Conclusion

In human salivary gland progenitor cells, TGF-β1 can induce K7 expression in a SMAD 2/3 dependent manner, which is suppressible by a TGFβR inhibitor. When hS/PCs are encapsulated in RGDSP presenting hydrogels, SMAD 2/3 signaling proceeds independently of nuclear YAP expression and is maintained by TGF-β1 levels. Expression of K7 by adult salivary gland cells is a TGF-β1 dependent stress response that can involve features of EMT.

## Experimental

### Materials

Reagents were procured from Fisher Scientific and used as received unless otherwise noted.

### Cell isolation and maintenance

hS/PCs were isolated from human salivary gland tissue, and cultured following reported procedures^45,46^. Parotid gland biopsies were obtained patients in agreement with protocols approved by institutional review boards at Christiana Care and the University of Delaware. Informed consent was obtained from all subjects and/or their legal guardians and all methods were performed in accordance with the relevant guidelines and regulations. hS/PCs were maintained in HepatoSTIM medium (355056; Corning Inc., Corning, NY) supplemented with 100 U mL^−1^ penicillin–streptomycin, 1% (v/v) amphotericin B, and 10 ng mL^−1^ epidermal growth factor (EGF). Passaging was conducted at 70–80% confluence using 0.05% (w/v) trypsin–EDTA. Trypsin was neutralized using a trypsin soybean inhibitor (T6522; Sigma Aldrich, St. Louis, MO). Experiments were conducted with at least three different donors at passages between 3–4.

### Hydrogel synthesis

Thiolated and acrylated HA derivatives (HA-SH and HA-AES) and maleimide-functionalized RGDSP (MI-RGDSP) were synthesized as previously reported^46,47,96,97^.

### 3D encapsulation

HA-SH was reconstituted in sterile PBS at 1% (w/v) to prepare HA hydrogels. To prepare RGDSP hydrogels, a 0.25 mM solution of MI-RGDSP in PBS was sterilized by filtration through a 0.2 μm Acrodisc PTFE syringe filter (4602; Pall Corporation, Port Washington, NY) and combined with the HA-SH solution. The resulting HA-SH or HA-SH/RGDSP solutions were neutralized to pH 7.4 using sterile 0.1 M NaOH, and hS/PC cell pellets, targeting a final cell concentration of 1 × 10^6^ cells mL^−1^ were suspended in these solutions by gentle pipetting. The HA-SH containing cell suspensions were combined with HA-AES, 1% (w/v) in PBS, at a 1/20 (v/v) ratio to initiate crosslinking. Media was refreshed every 72 h and cultures were terminated on day 14. Characterization of protein and mRNA expression was conducted using 100 μL hydrogels prepared in 12 mm Millicell 04 μm PTFE cell culture inserts (PICM01250; EMD Millipore) with 800 μL HeptoSTIM media. ICC characterization was conducted using 30 μL hydrogels cultured in 48-well uncoated glass-bottom plates with 1.5 coverslip (P48G-1.5-6-F; MatTek, Ashland, MA) containing 300 μL media. When A83-01 inhibition was performed, HeptoSTIM media supplemented with 2 μM A83-01 or the vehicle control (DMSO) media were exchanged every 48 h.

### 2D hS/PC culture and characterization

hS/PCs were seeded at 35,000 cells cm^−3^ and cultured for 48 h before introducing the indicated growth factors and inhibitors. For ICC studies, cells were cultured on 8-well Nunc Lab-Tek II chambered coverglass (155409; Thermo Scientific, Waltham, MA). For gene expression studies, cells were cultured in Nunc cell-culture treated 6-well plates (140675; Thermo Scientific). HepatoSTIM medium was supplemented with 10 ng mL^−1^ of recombinant human TGF-β1 (100-21; PeproTech, Rocky Hill, NJ), 100 ng mL^−1^ of recombinant human GDF-15 (120-28C; PeproTech), and 2 µM of A83-01 (9001799; Cayman Chemical Company, Ann Arbor, MI) as a single factor or in combination when appropriate. ICC studies involving verteporfin (VERT, SML0534; MilliporeSigma) were conducted at 0.5, 1.0, and 2.0 µM in complete darkness^98–100^, and experiments were terminated after 24 h of culture. Gene expression studies were performed with VERT at 1.0 µM. In all cases, vehicle controls were maintained using PBS, DMSO, or a combination thereof, as required. RNA isolation was conducted with TRIzol and purified with RNA Clean & Concentrator-5 Kit as described in the 3D isolation protocol.

### 3D immunofluorescence

Hydrogel constructs were fixed with 4% (w/v) paraformaldehyde (PFA) in PBS for 1 h. PBS was supplemented with 500 U mL^−1^ penicillin–streptomycin and 0.05% (v/v) sodium azide to produce PBS + PS, which was combined with the permeabilization agent detailed in Table S2 to prepare PBS/perm. Blocking was conducted for 16 h at 4 °C with 3% (w/v) bovine serum albumin (BSA) reconstituted in PBS/perm (BSA/perm). Primary antibodies were diluted in BSA/perm, and a 48-h incubation was performed on the fixed constructs. Hydrogel constructs were then washed with PBS/perm that was exchanged 5 times over 24 h. Alexa Fluor AffiniPure Fab Fragment Anti IgG (H + L) secondary antibodies (Jackson Immunoresearch Labs, West Grove, PA) were diluted at 1/200 (v/v) in the BSA/perm solution, and constructs were subsequently incubated in the secondary antibody solution for 48 h at room temperature. Hydrogel constructs were then washed with PBS/perm that was exchanged 3 times over 3 h. Next, 4′,6-diamidino-2-phenylindole (DAPI, D1306; Life Technologies, Carlsbad, CA) and Alexa Fluor 568 Phalloidin (phalloidin, A12380; Life Technologies, Carlsbad, CA) were diluted at 1/1000 and 1/450 (v/v) respectively in PBS/perm. Constructs were incubated in DAPI and phalloidin solutions for 12 h at room temperature. The hydrogel constructs were then washed for 6 h with PBS/perm that was exchanged 3 times. ICC incubation and wash steps were conducted using an orbital shaker at 250 RPM. Mounting was conducted by incubating for 16 h at 4 °C with VECTASHIELD PLUS Antifade Mounting Medium (H-1900; Vector Laboratories, Burlingame, CA). Extended information detailing primary antibody, secondary antibody, and permeabilization agent can be found in Table S2.

Fluorescent microscopy was performed with a Zeiss LSM 880, ZEN 2.3 SP1, equipped with an Airy scan super-resolution detector (Carl Zeiss, Oberkochen, Germany). An LD LCI Plan-Apochromat 25×/0.8 Imm Korr objective was used with Immersol 518F (Carl Zeiss) using a refractive index 1.518. Image acquisition was performed using Fast Airyscan mode, and a piezoelectric stage was used to sequentially capture single-channel excitations along the z-axis. Volumetric images were recorded with an x–y area of 83.54 µm and 0.492 µm z-axis scaling. Fast Airyscan processing was performed with ZEN 3.0 SR software producing 16-bit images, and SMAD 2/3 and YAP expression was quantified using Imaris 9.7.0 3D-4D Imaging Software (Oxford Instruments, Abingdon, United Kingdom).

Following scientific digital image ethical guidelines^101,102^, representative hS/PC structures were centered to the field of view by cropping the x–y area to 63.39 µm before uniformly applying brightness adjustments with ZEN 3.0 SR software. To minimize power variation and laser instability during quantitative imaging, imaging was completed within 3 sessions, and lasers were warmed for a minimum of 30 min prior to the start of an image session^101^.

### 2D immunofluorescence

hS/PC cultures were terminated with PFA for 30 min. PBS + PS was used to reconstitute BSA with the appropriate permeabilization agent as detailed in Table S4 to prepare PBS/perm. Blocking was conducted for 16 h at 4 °C with the indicated (Table S4) PBS/perm solutions. Primary and highly cross-adsorbed whole goat IgG Alexa Fluor (A-11029, A-21236, A-11034, A-21245; Invitrogen) secondary antibodies were incubated, sequentially, for 16 h at 4 °C followed by 1 h at room temperature. Secondary antibodies were diluted at 1/250 in all cases. Following antibody incubations, samples were washed with PBS/perm that was exchanged 6 times over 6 h at room temperature. Next, DAPI and phalloidin were diluted at 1/1000 and 1/450 (v/v) respectively in PBS/perm and incubated with samples for 90 min at room temperature. Samples were mounted with VECTASHIELD PLUS antifade mounting medium. Extended information detailing primary antibody, secondary antibody, and permeabilization agent can be found in Table S4. Fluorescent microscopy was conducted with a Zeiss LSM 880, ZEN 2.3 SP1, fitted with an Airy scan super-resolution detector. Imaging conducted with C-Apochromat 10×/0.45 W or LD LCI Plan-Apochromat 25×/0.8 Imm Korr objectives were used with deionized water or Immersol 518F (Carl Zeiss) respectively. Image acquisition was conducted using Fast Airyscan mode and with sequential channel capture, frame mode. Fast Airyscan processing was performed with ZEN 3.0 SR software, generating 16-bit images, and quantification of protein expression and localization was assessed with ImageJ. Binary nuclear images were produced by applying a moments threshold to the DAPI channel, then a watershed segmentation and adding to the ImageJ ROI manager using the magic wand tool. The corresponding cell bodies were isolated by manually tracing the F-actin channel using the segmented line tool and adding the selection to the ROI manager. A Huang threshold was applied to the SMAD 2/3 channel, and individual cell measurements were made using the limit to threshold function. Cytoplasmic expression was determined by subtracting the integrated density (ID) of nuclear measurements from the corresponding cell body ID measurements. ID is reported after background corrections were applied using the relationship (ID = area × mean gray value) and normalizing to the mean ID of non-treated controls from each biological experiment. K7 expression was analyzed similarly as above; segmentation was performed by manually tracing the F-actin channel using the segmented line tool, and a Huang threshold was applied to the K7 channel. Brightness and contrast adjustments were uniformly applied to the selected representative images using ZEN 3.0 SR software. A blinded reviewer performed analysis, and a minimum of 60 measurements was made for each condition. β-catenin intensity was analyzed using the ImageJ line profile tool.

### Immunoblot array

Expression of TGF-β family members was investigated using a Human TGF beta Array C2 (AAH-TGFB-2-2; RayBiotech, Peachtree Corners, GA). The array template is shown in Fig. S2c. Media collected from the cellular constructs on day 6 and 14 was separately pooled from three biological replicates to meet the minimum volume requirements and used to carry out the assay following the manufacturer’s procedure. Chemiluminescent signals were recorded using an iBright FL1500 Imaging System. Image processing was performed using ImageJ following the manufacturer’s protocol, and values were then normalized to the duration of culture.

### Enzyme linked immunosorbance assay (ELISA)

Medium was collected from hydrogel cell constructs on days 3, 6, 9, and 12 of culture and stored at − 80 °C until analysis. Total TGF-β1 levels were assessed using Human TGF-beta 1 DuoSet ELISA (DY240; R&D Systems, Minneapolis, MN). Similarly, GDF-15 expression was analyzed using Human GDF-15 DuoSet ELISA (DY957; R&D Systems). For the activation of latent TGF-β1, the medium was treated with Sample Activation Kit (DY010; R&D Systems). TGF-β1 and GDF-15 ELISA were performed using Substrate Reagent Pack (DY999; R&D Systems), and absorbance measurements were made at 450 nm with a 570 nm correction using a SpectraMax i3x Multi-Mode Microplate Reader. α-Amylase expression was quantified using the Human Salivary Amylase Alpha ELISA Kit (NBP2-68203; Novus Biologicals, Littleton, CO), and luminesce measurements were made using a SpectraMax i3x Multi-Mode Microplate Reader. All assays were performed in accordance with the manufacturers’ protocols. Protein levels were normalized to the number of cells in each hydrogel at each time point using the procedure detailed in the 3D Proliferation method conducted on days 1, 3, 6, 9, and 12 of culture.

### Simultaneous RNA, DNA, and protein isolation from 3D cultures

Isolation of protein and nucleic acids from the same experimental sample was conducted using TRIzol™ (15596026; Invitrogen, Carlsbad, CA) and adapting the manufacture’s recommended procedure. Detailed methodology of nucleic acid and protein extraction is included in the supporting information. Additional information regarding RT-qPCR, Quant-iT PicoGreen dsDNA Assay Kit (P11495; Invitrogen), and western blot assay conducted with chemifluorescencent detection (ECF) are included in the supporting information.

### Statistical analysis

Comparisons between two experimental groups were made using a two-tailed Student’s t-test. A one-way analysis of variance (ANOVA) was performed when three or more experimental groups were compared, and a two-way ANOVA was performed when data sets included an additional variable. When ANOVAs returned an F statistic greater than F critical, a post-hoc Tukey multiple comparisons test was carried out. For multiple comparisons containing a control group, a post-hoc Dunnett's test was performed. *p* < 0.05 was deemed significant and indicated by *,^†^,^‡^, or ^§^. Significance from day 1, 3, 6 and 7, 9 and 12 samples of the same experimental group are indicated by ^†^,^‡^, and ^§^ respectively. Statistical interpretations were made using JMP Pro 15 (SAS Institute Inc., Cary, NC).

## Supplementary Information

Supplementary Information.

## Data Availability

The datasets generated and/or analysed during the current study are not publicly available as it is part of an ongoing study but are available from the corresponding author on reasonable request.
