# Supplementary material for: A TGFβR inhibitor represses keratin-7 expression in 3D cultures of human salivary gland progenitor cells
Source: Sci Rep. 2022 Sep 2;12:15008. doi: 10.1038/s41598-022-19253-x (PMC9440137; doi:10.1038/s41598-022-19253-x)
Supplement: Supplementary file 1 — Supplementary Information. [file 41598_2022_19253_MOESM1_ESM.pdf]

Supporting Information

A TGFβR Inhibitor Represses Keratin-7 Expression in 3D Cultures of Human Salivary Gland Progenitor Cells

Eric W. Fowler,[a,] Emmett J. van Venrooy,[b] Robert L. Witt,[c] Xinqiao Jia[a,b,d,e,]**

[a] Department of Materials Science and Engineering, University of Delaware, Newark, DE, 19716, USA

[b] Department of Biological Sciences, University of Delaware, Newark, DE, 19716, USA

[c] Helen F. Graham Cancer Center and Research Institute, Christiana Care, Newark, DE, 19713, USA

[d] Department of Biomedical Engineering, University of Delaware, Newark, DE, 19716, USA

[e] Delaware Biotechnology Institute, 590 Avenue 1743, Newark, DE 19713, USA

*To whom correspondence should be addressed:

Eric W. Fowler, Ph.D., Department of Materials Science and Engineering, University of Delaware, Newark, DE, 19716, USA. Phone: (302) 831-2401, Email: fowlere@udel.edu

Xinqiao Jia, Ph.D., Department of Materials Science and Engineering, University of Delaware, Newark, DE, 19716, USA. Phone: (302) 831-6553, E-mail: xjia@udel.edu

Keywords: Salivary Gland, Hydrogels, RGDSP, Keratin-7, TGF-β1, SMAD 2/3, YAP

Simultaneous RNA, DNA and Protein Isolation from 3D Cultures. On days 1, 3, 7, and 14 of culture, hS/PC laden HA hydrogel constructs were frozen with liquid nitrogen and stored at -80 °C. Extractions were conducted by crushing the frozen hydrogel constructs with polypropylene Pellet Pestles (12-141-367; Fisher Scientific), followed by treatment with 750 µL TRIzol reagent. After complete homogenization in TRIzol, the samples were centrifuged at 4 °C for 5 min at 15,000 × g. The resulting pellet with insoluble cross-linked HA hydrogel was temporarily stored at 4 °C for subsequent isolation of DNA and protein. The TRIzol supernatant was collected, and chloroform (150 µL) was added before incubating the samples for 3 min at room temperature. The supernatant was then centrifuged at 4 °C for 20 min at 15,000 × g to yield a phase-separated solution. The lower phase was collected for DNA and protein isolation and temporarily stored at 4 °C. The upper aqueous phase was collected, and RNA purification was conducted using an RNA Clean & Concentrator-5 Kit (R1013; Zymo Research, Irvine, CA) according to the manufacturer's protocol. After column elution, RNA quantification and purity were assessed using a NanoDrop 2000 Spectrophotometer (Nanodrop Technologies, Wilmington, DE). This technique yielded high purity RNA with absorbance ratios 260/280 nm >1.95 and 260/230 >1.8.

To continue the DNA isolation, the insoluble fraction generated from the initial TRIzol homogenized HA hydrogel was recombined with the corresponding lower TRIzol phase and briefly vortexed to permit dissolution. Ethanol (100%, 2.5 mL) was then added, and samples were mixed by inversion, incubated at room temperature for 3 min, then centrifuged at 2000 × g at 4 °C for 5 min. The phenol-ethanol supernatant was collected and stored at 4 °C until further protein isolation described below. The resulting pellet was incubated with 750 µL of 0.1 M sodium citrate containing 10% (v/v) ethanol and mixed by gentle inversion for 30 min at room temperature. The DNA pellet was centrifuged at 4 °C for 5 min at 2,000 × g, and the procedure was conducted for a total of three treatments of the solution containing 0.1 M sodium citrate/10% (v/v) ethanol. The DNA pellet was dissolved in pH 9.5 TE buffer and stored at -80 °C until further characterization.

To continue the protein isolation, the phenol-ethanol supernatant was combined with isopropanol (1.5 mL) and incubated for 1 h at room temperature before an extended incubation was conducted for 16 h at 4 °C. Samples were then centrifuged at 4 °C for 1 h at 15,000 × g. The resulting protein pellet was then incubated at room temperature for 20 min with 0.3 M guanidine hydrochloride (GdnHCl) in 95% (v/v) ethanol (1.8 mL) before centrifuging at 4 °C for 20 min at 15,000 × g. Two additional GdnHCl washes were performed before washing with 100% ethanol. The protein pellets were next air-dried before dissolving in a solution of Tris HCl (100 mM, pH 8.0), prepared with 4 M urea, 5% (w/v) sodium dodecyl

sulfate, 140 mM NaCl, 20 mM ethylenediaminetetraacetic acid, and 10% (v/v) glycerol, then stored at -20 °C until further characterization.

dsDNA Quantification. DNA solutions obtained via TRIzol extraction were centrifuged at 12,000 × g for 10 min at 4 °C. The resulting supernatant was analyzed using a Quant-iT PicoGreen dsDNA Assay Kit following the manufacturer's protocol. Fluorescent measurements were conducted using a SpectraMax i3x Multi-Mode Microplate Reader (Molecular Devices, San Jose, CA) with a 485 nm excitation and 520 nm emission wavelength.

Western Blotting. The protein concentration of cell lysates, obtained by TRIzol extraction, were quantified using a Micro BCA Protein Assay Kit (23235; Thermo Scientific, Waltham, MA), according to the manufacturer's procedure, and measurements were performed on a SpectraMax i3x Multi-Mode Microplate Reader at 562 nm. The cell lysate was then combined with 3 × Blue Loading Buffer (56036; Cell Signaling Technology, Danvers, MA; 187.5 mM Tris-HCl, pH 6.8, 6% (w/v) SDS, 30% glycerol and 0.03% (w/v) bromophenol blue) and prepared with 0.2 M DTT (7016L; Cell Signaling Technology). Next, samples were incubated for 5 min at room temperature before heating at 95 °C for 10 min and vortexed for 30 s before centrifuging at 15,000 × g for 2 min at room temperature. SDS-PAGE was conducted using 4–20% Mini-PROTEAN TGX Stain-Free Protein Gels (4561093; Bio-Rad Laboratories, Hercules, CA) incubated with Tris-Glycine-SDS buffer (1610732; Bio-Rad Laboratories). Sample lysate was loaded at 10 µg per lane and applied with 40 volts for 30 min followed by 300 volts for 20 min at 4 °C. Samples were transferred to nitrocellulose membranes (12369P2; Cell Signaling Technology) at 4 °C for 2 h using 70 volts. For protein lane normalization, the nitrocellulose membranes were treated with Revert 700 Total Protein Stain (926-11010; LI-COR Biosciences, Lincoln, NE) according to the manufacturer's protocol and imaged using an iBright FL1500 Imaging System (Invitrogen, Carlsbad, CA). Membranes were blocked with 5% (w/v) skim milk (sm/TBST) in tris-buffered saline containing 0.1% Tween 20 (TBST, 9997; Cell Signaling Technology) for 2 h at room temperature. Primary antibodies (Table S3) were then diluted in sm/TBST and incubated for 16 h at 4 °C. Membranes were next washed three times with TBST for 5 min at room temperature. Alkaline phosphatase-linked secondary antibodies (Cell Signaling Technology, Table S3) were diluted 1/20,000 in sm/TBST and incubated for 1 h at room temperature before washing with TBST three times at room temperature. Chemifluorescence signal was introduced using Cytiva Amersham ECF substrate (RPN5785; MilliporeSigma) and visualized using an iBright

FL1500 Imaging System. Densitometry analysis was conducted with ImageJ using the analyze gels feature, in agreement with the procedure outlined in the ImageJ documentation. (https://imagej.nih.gov/ij/docs/menus/analyze.html#gels)

qPCR Assay. RNA was reverse transcribed using the QuantiTect Reverse Transcription Kit (205314; QIAGEN, Hilden, Germany) following the manufacturer's protocol. Sequence-specific amplification and detection was performed on an ABI 7300 real-time PCR system (Applied Biosystems, Foster City, CA), with a thermal cycling profile of 1 cycle at 95 °C for 10 min, followed by 40 cycles of 95 °C for 15 s and 60 °C for 1 min. Twenty µL PCR reactions were prepared in 96 well format by combining Power SYBR green PCR master mix (Applied Biosystems), cDNA, and target specific primers. Primer designs were primarily sourced from PrimerBank,[1] (https://pga.mgh.harvard.edu/primerbank/) and the complete primer sequences are available in Table S1. Primers were synthesized by Integrated DNA Technologies (Coralville, IA) and utilized after confirming to amplify a single target on agarose gels with an amplicon size corresponding to that determined from Primer-BLAST.[2,3] Glyceraldehyde 3-phosphate dehydrogenase (GAPDH) was used as a reference gene, and inter-run calibrations were included when gene expression was examined across multiple time points.[4] Cycle threshold values were generated using 7300 System SDS RQ Study software version 1.4 (Applied Biosystems), and mRNA levels were resolved using Qbase+ software version 3.2 (Biogazelle, Zwijnaarde, Belgium). Three biological replicates are reported from three technical replicates measured in duplicate.

Validation of Fluorescent Intensity Measurements. Pilot studies were performed to confirm that quantitative fluorescent intensity measurements were valid at distances, z-dimension, up to 500 µm into the hydrogel constructs, and image collection was limited to this distance. Briefly, Alexa Fluor 647 labeled reference standards (BLI887A-1; Polysciences, Warrington, PA) were encapsulated in HA hydrogels, and z-stack images were collected from the coverslip to the working distance of the piezoelectric stage. Imaris Imaging Software was used to derive the reference standard mean intensities, and their corresponding distance from the coverslip was fit to a linear model (Fig S1).

3D Cell Proliferation. hS/PC proliferation in 3D was determined by enumerating nuclei in confocal z-stack images collected on days 1, 3, 7, and 14 of culture. Cell-laden hydrogel constructs were fixed with 4% PFA at the designated time points, and nuclei were labeled with Hoechst 3342 (Hoechst, ThermoFisher

Scientific) at 10 μg mL^{-1} in PBS for 30 min. Microscopy was conducted with a Zeiss LSM 880 equipped with an Airyscan detector using a 10×C-Apochromat 0.45 N.A water immersion objective. Images were captured as 300.45 μm z-stacks with a 1.757 μm z-axis step size and x-y area of 850.19 μm, and fast airy scan processing was performed using Zen Black 3.0 SR. Nuclei were resolved and counted using 3D-4D Imaging Software Imaris 9.7.0. Three independent experiments were conducted at each time point.

Table S1. Primers used for qPCR assays.

Target	Forward Primer (5'-3')	Reverse Primer (5'-3')
GAPDH	CAGCCTCAAGATCATCAGCA	TGTGGTCATGAGTCCTTCCA
KRT5	CGTGCCGCAGTTCTATATTCT	ACTTTGGGTTCTCGTGTCAG
KRT14	CACAGATCCCACTGGAAGAT	GATAATGAAGCTGTATTGATTGCC
TFCP2L1	GCCGCCTGCTTCCTGTTC	CTGCCCACCACTGCTCAAAG
AMY1A	CTCGGCACAGTTATTCGCAAGTGG	ACAGCCTAGCATCCCAGAAGGT
SLC12A2	TAAAGGAGTCGTGAAGTTTGGC	CTTGACCCACAATCCATGACA
KRT7	AAGAACCAGCGTGCCAAGT	TCCAGCTCCTCCTGCTTG
KRT18	GTTGACCGTGGAGGTAGA	GACCCAGCTCGTCATATTGGG
KRT19	CTGCCTCCAAGGTCCTCT	CCCATCCCTCTACCCAGAAG
KRT7-AS	TCCAACGCCTATGTTCCAGTTC	ACATTGTGCCACGGACATCTTG
TP53	CAGCACATGACGGAGGTTGT	TCATCCAAATACTCCACACGC
TGFB1	GCAGAAGTTGGCATGGTAGC	CCCTGGACACCAACTATTGC
GDF15	GACCCTCAGAGTTGCACTCC	GCCTGGTTAGCAGGTCCTC
YAP1	ACCCACAGCTAGCATCTTCG	TGGCTTGTTCCCATCCATCAG
CTGF (CCN2)	AGGAGTGGGTGTGTGACGA	CCAGGCAGTTGGCTCTAATC
CYR61 (CCN1)	CCTTGTGGACAGCCAGTGTA	ACTTGGGCCGGTATTTCTTC
CCND1	TGGAGCCCGTGAAAAAGAGC	TCTCCTTCATCTTAGAGGCCAC
JUNB	ACGACTCATACACAGCTACGG	GCTCGGTTTCAGGAGTTTGTAGT
SMAD3	TGGACGCAGGTTCTCCAAAC	CCGGCTCGCAGTAGGTAAC
MYC	CGGAACTCTTGTGCGTAAGG	TCATAGGTGATTGCTCAGGACAT
EREG	GTGATTCCATCATGTATCCCAGG	GCCATTCATGTCAGAGCTACACT
AREG	GTGGTGCTGTCGCTCTTGATA	CCCCAGAAAATGGTTCACGCT
HBEGF	ATCGTGGGGCTTCTCATGTTT	TTAGTCATGCCCAACTTCACTTT
TGFA	AGGTCCGAAAACACTGTGAGT	AGCAAGCGGTTCTTCCCTTC
ADAM10	ATGGGAGGTCAGTATGGGAATC	ACTGCTCTTTTGGCACGCT

ADAM17	GTGGATGGTAAAAACGAAAGCG	GGCTAGAACCCTAGAGTCAGG
TIMP3	CATGTGCAGTACATCCATACGG	CATCATAGACGCGACCTGTCA
TIMP1	TTTCTTGGTTCCCCAGAATG	CAGAGCTGCAGAGCAACAAG
MMP1	GGGAGATCATCGGGACAACTC	GGGCCTGGTTGAAAAGCAT
FN	ACCTACGGATGACTCGTGCTTTGA	CAAAGCCTAAGCACTGGCACAACA
LAMA1	GTGATGGCAACAGCGCAAA	GACCCAGTGATATTCTCTCCCA
ITGA5	GCCTGTGGAGTACAAGTCCTT	AATTCGGGTGAAGTTATCTGTGG
ITGAV	AATCTTCCAATTGAGGATATCAC	AAAACAGCCAGTAGCAACAAT
IL6	ACTCACCTCTTCAGAACGAATTG	CCATCTTTGGAAGGTTCAGGTTG
CXCL8	ACATACTCCAAACCTTTCACCC	CAACCCTCTGCACCCAGTTTTC
IL10	GGTTGCCAAGCCTTGTCTGA	AGGGAGTTCACATGCGCCT
IL1B	GCTGATGGCCCTAAACAGATGA	TTGCTGTAGTGGTGGTCGGAGAT
CDKN1A (P21)	TGTCCGTCAGAACCCATGC	AAAGTCGAAGTTCCATCGCTC
CDKN2A (P16)	GATCCAGGTGGGTAGAAGGTC	CCCCTGCAAACTTCGTCCT
SERPINE1	ACCGCAACGTGGTTTTCTCA	TTGAATCCCATAGCTGCTTGAAT
JUNB	ACGACTCATACACAGCTACGG	GCTCGGTTTCAGGAGTTTGTAGT
IGF1	GCTCTTCAGTTCGTGTGTGGA	GCCTCCTTAGATCACAGCTCC
IGF2	GTGGCATCGTTGAGGAGT	CACGTCCCTCTCGGACTT
IGF1R	TCGACATCCGCAACGACTATC	CCAGGGCGTAGTTGTAGAAGA

Table S2. Antibodies and conditions used for 3D immunocytochemistry.

Target	Vendor	Clone/ Item No./RRID	Host	Perm (w/v)	Dilution (v/v)	Secondary Antibody
YAP1	Santa Cruz Biotechnology	(63.7)/ sc-101199 RRID:AB_1131430	Ms	0.2% Triton	1/50	Alexa Fluor 647 AffiniPure Fab Fragment Goat Anti-Ms IgG (H+L) AB_2338931
SMAD 2/3	Cell Signaling Technology	(D7G7) XP/ 8685S RRID:AB_10889933	Rb	0.2% Triton	1/100	Alexa Fluor 488 AffiniPure Fab Fragment Goat Anti-Rb IgG (H+L) AB_2338058
α-Amylase	MilliporeSigma	A8273/ RRID:AB_258380	Rb	0.05% Saponin	1/100	Alexa Fluor 488 AffiniPure Fab Fragment Goat Anti-Rb IgG (H+L) AB_2338058

Table S3. Antibodies and conditions used for western blotting.

Target	Vendor	Clone/ Item No./RRID	Host	Dilution (v/v)	Secondary Antibody
Keratin 5	BioLegend	905501 RRID:AB_2565050	Rb	1/20,000	Anti-rabbit IgG, AP-linked Antibody #7054[i] RRID:AB_2099235
Fibronectin	MilliporeSigma	F3648 RRID:AB_476976	Rb	1/1000	Anti-rabbit IgG, AP-linked Antibody #7054[i] RRID:AB_2099235
Keratin 7	MilliporeSigma	OV-TL 12/30/ MAB3554 RRID:AB_94924	Ms	1/1000	Anti-mouse IgG, AP-linked Antibody #7056[i] RRID:AB_330921
Keratin 14	Abcam	LL002/ Ab7800 RRID:AB_306091	Ms	1/1000	Anti-mouse IgG, AP-linked Antibody #7056[i] RRID:AB_330921

(i) Cell Signaling Technology

Table S4. Antibodies and conditions used for 2D immunocytochemistry.

**Target**	**Vendor**	**Clone/ Item No./RRID**	**Host**	**Perm (w/v)**	**Dilution (v/v)**	**Secondary Antibody**
YAP1	Santa Cruz Biotechnology	(63.7)/ sc-101199 RRID:AB_1131430	Ms	0.2% Triton	1/50	Alexa Fluor 647 Goat anti-Ms RRID:AB_2535805
SMAD 2/3	Cell Signaling Technology	(D7G7) XP/ 8685S RRID:AB_10889933	Rb	0.2% Triton	1/100	Alexa Fluor 488 Goat anti-Rb RRID:AB_2576217
Keratin 5	BioLegend	905501 RRID:AB_2565050	Rb	*0.05% Saponin	1/100	Alexa Fluor 647 Goat anti-Rb[ii] RRID:AB_2535813
Keratin 7	MilliporeSigma	OV-TL 12/30/ MAB3554 RRID:AB_94924	Ms	0.05% Saponin	1/50	Alexa Fluor 488 Goat anti-Ms RRID:AB_2534088
Ki-67	Invitrogen	7B11/ 334711/ RRID: AB_2533122	Ms	0.2% Triton	1/450	*NA* (FITC conjugated)

(ii) K5/YAP1 ICC was performed with 0.2 % Triton (w/v) and Alexa Fluor® 488 goat anti-Rb

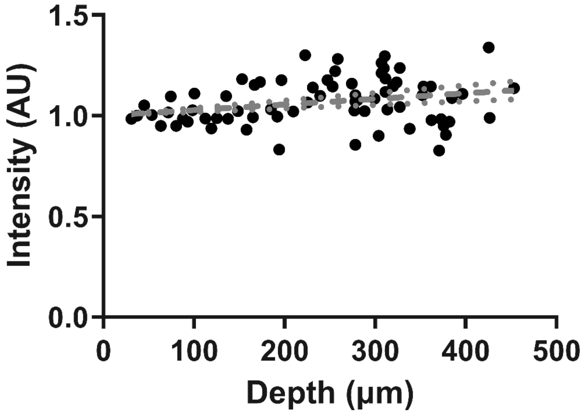

Fig. S1. Alexa Fluor 647 labeled reference microsphere standards were suspended in HA hydrogels, and z-stack images were captured with fluorescent microscopy. The mean intensity of individual microspheres (black filled circles) was determined using Imaris 3D-4D imaging software. Linear regression of microsphere normalized mean intensity calculated using GraphPad Prism 9 (GraphPad Software, San Diego, CA) is shown as gray dotted line; gray dots represent 95% confidence intervals.

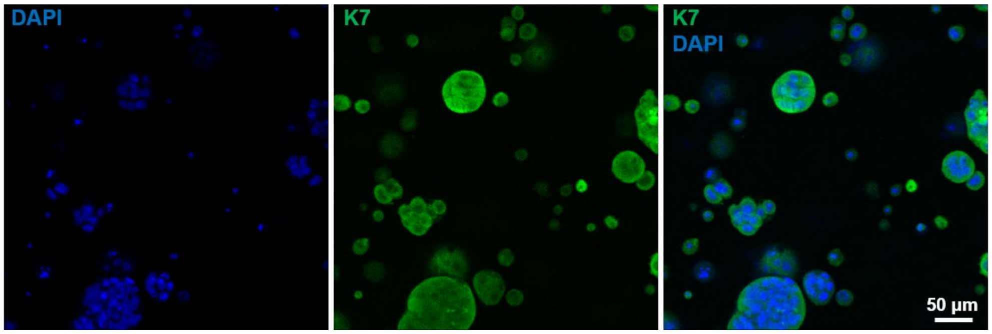

Fig. S2. hS/PCs were cultured in RGDSP constructs for 14 days, and ICC was conducted to visualize K7 (green) expression by fluorescent microscopy. Cell nuclei were counter stained in blue.

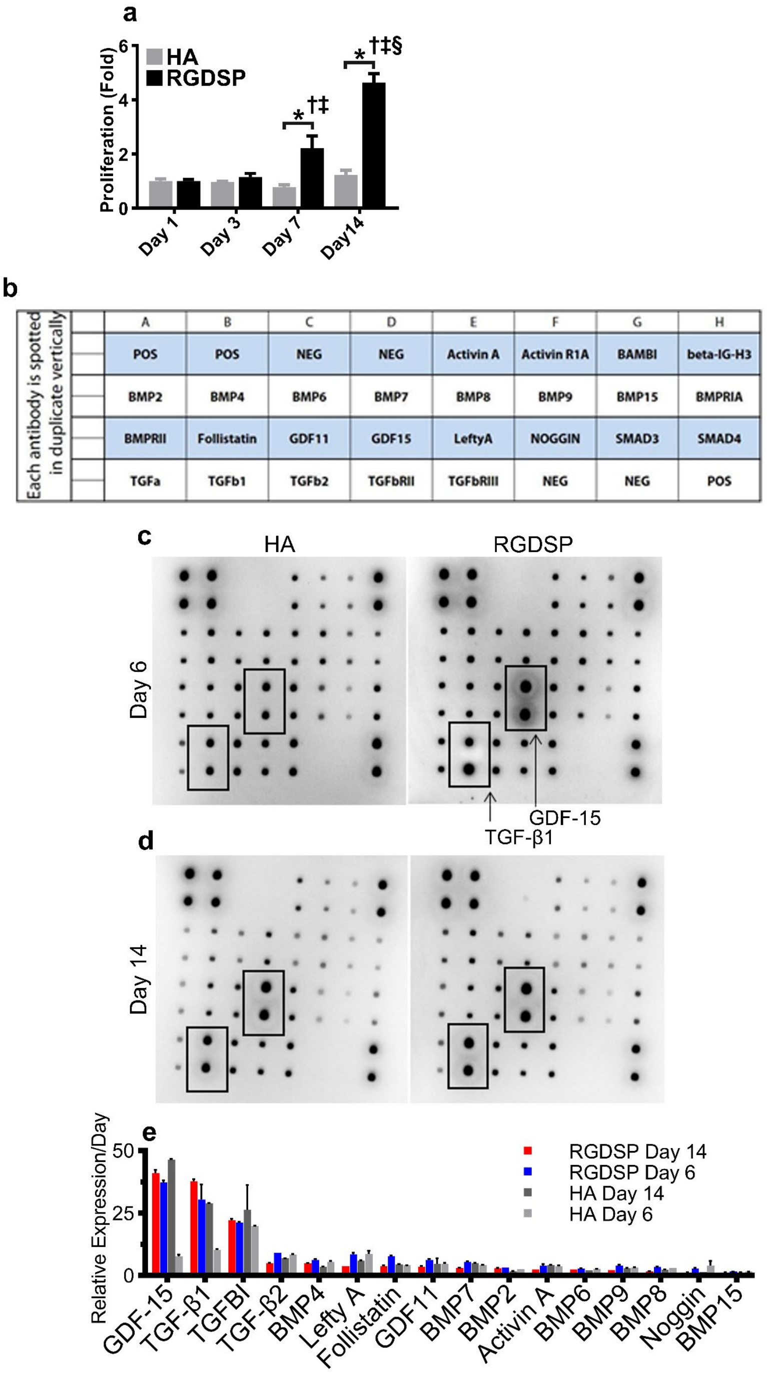

Fig. S3. (**a**) hS/PCs were cultured in HA and RGDSP hydrogels and DAPI stained nuclei were recorded with fluorescent microscopy before cell density quantification was performed with Imaris 3D Software; two-way ANOVA was performed followed by Tukey's multiple comparisons test. * indicates $p < 0.05$ between HA and RGDSP at the same time point. †, ‡, § indicates $p < 0.05$ from day 1, 3, and day 7 measurements of the same data set, respectively. (**b**) TGF-β Superfamily immunoblot array template. (**c-d**) TGF-β superfamily immunoblot array performed on media collected on day 6 (c) and 14 (d). TGF-β Superfamily arrays were quantified with ImageJ and normalized to the duration of culture. Error bars represent SEM in all cases.

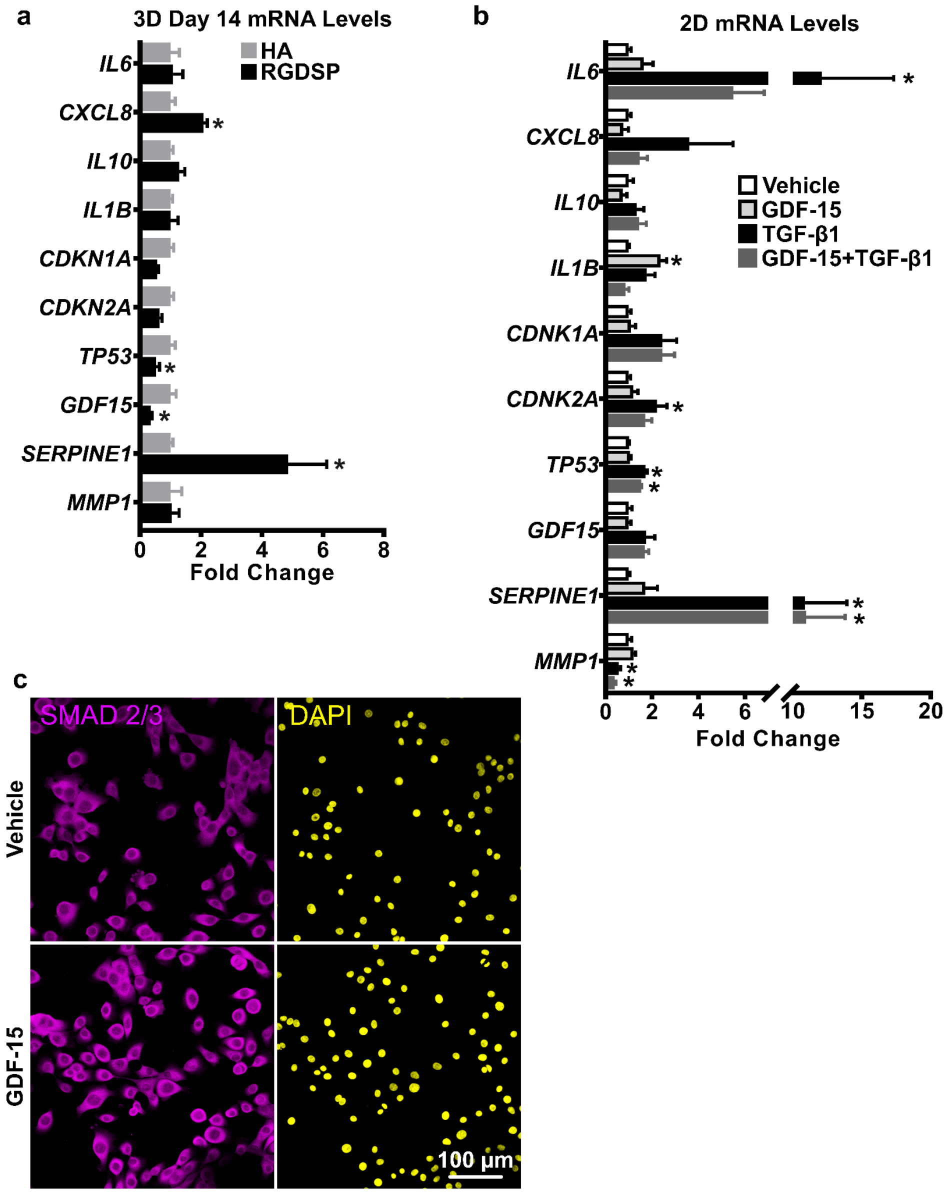

Fig. S4. (**a**) hS/PCs were cultured for 14 days in HA, and RGDSP constructs and the expression of SASP associated genes were assessed by qPCR; Student's t-test, * indicates $p < 0.05$. (**b**) 2D cultured hS/PCs were treated with TGF-β1, GDF-15, and GDF-15+TGF-β1 for 48 h before assessing expression of SASP associated genes; one way-ANOVA was performed followed by a Dunnett's test. * indicates $p < 0.05$. (**c**) hS/PCs were treated with GDF-15 for 48 h, and the expression of SMAD 2/3 was visualized with fluorescent microscopy. Error bars represent SEM in all cases.

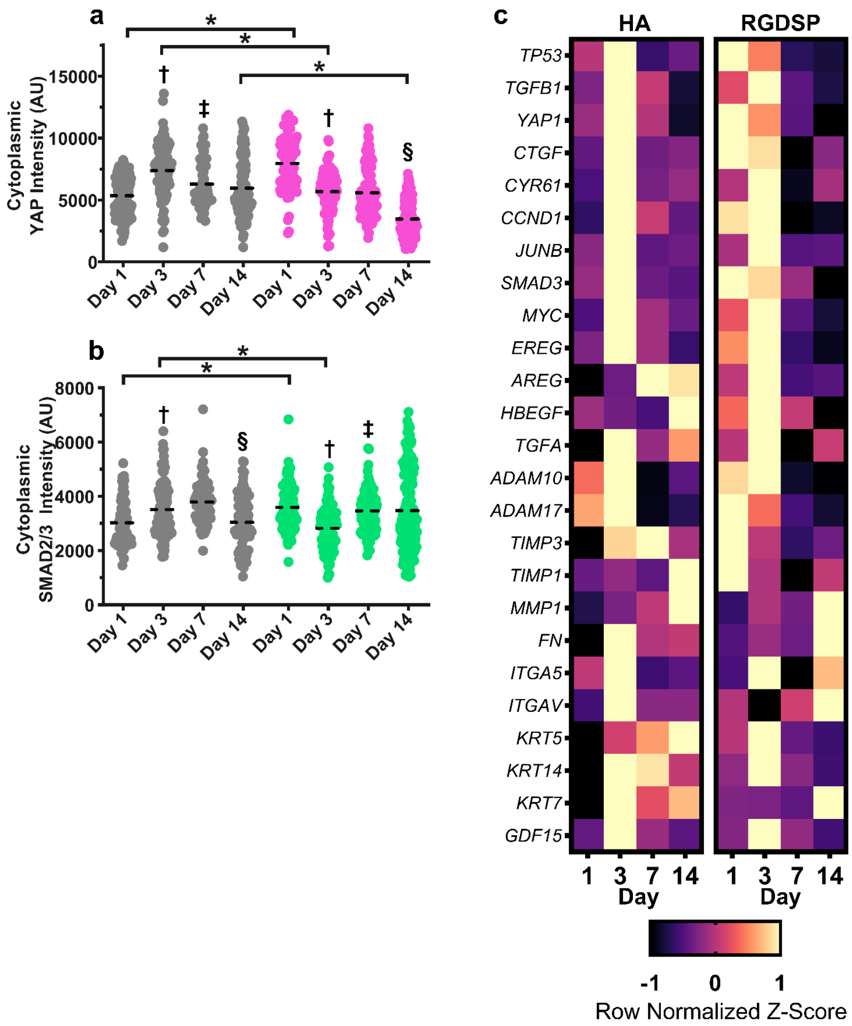

Fig. S5. (**a-b**) hS/PCs were cultured in HA and RGDSP, and the cytoplasmic expression of YAP (a) and SMAD 2/3 (b) was resolved *via* ICC. Filled circles represent individual cells or multicellular structures as spheroids develop, and the dashed black line indicates the mean value of each data set. n_{HA}= 104 (day 1), 105 (day 3), 66 (day 7), 113 (day 14); n_{RGDSP}=93 (day 1), 105 (day 3), 114 (day 7), 182 (day 14) conducted from 3 independent experiments. Two-way ANOVA was performed, followed by Tukey's multiple comparisons test. * indicates $p < 0.05$ between HA and RGDSP at the same time point. $^{\dagger, \ddagger, \S}$ indicates $p < 0.05$ from day 1, 3, and day 7 measurements of the same data set, respectively. (**c**) The expression of TGF-β and YAP target genes, *TP53,*[5] *TGFB1,*[6] *YAP1,*[7] *CTGF,*[7-9] *CYR61,*[7,8] *CCND1,*[7] *JUNB,*[10] *SMAD3,* [11] [12] *MYC,*[13] *EREG,*[8] *AREG,*[14,15] *HBEGF,*[15] *TGFA,*[15] *ADAM10,*[16] *ADAM17,*[16] *TIMP3,*[17] *TIMP1,*[17,18] *MMP1,*[18,19] *FN,*[12,20] *ITGA5,* [21,22] *ITGAV,*[12,22,23] *KRT5,*[8] *KRT14,*[8,9] and *GDF15,*[24] was assessed by qPCR on days 1, 3, 7, and 14 of HA and RGDSP cultures. Z-score normalization was performed on individual genes. Results were averaged from 3 independent experiments, each with 3 technical replicates (n = 9). Error bars represent SEM in all cases.

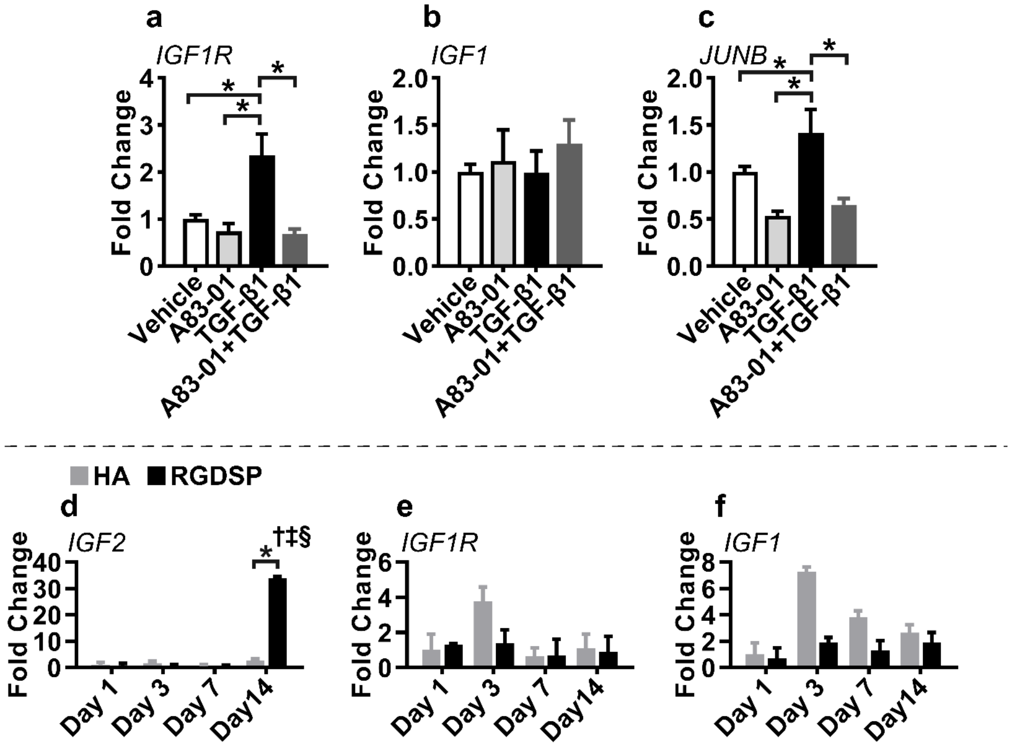

Fig. S6. (**a-c**) hS/PC were cultured with TGF-β1, A83-01, or in combination for 48 h before the expression of *IGF1R* (a), *IGF1* (b), and *JUNB* (c) was assessed with qPCR; n=9, 3 independent experiments, each with 3 technical replicates. One way-ANOVA was performed followed by Tukey's multiple comparison test. * indicates $p < 0.05$. (**d-f**) hS/PCs were cultured in HA, and RGDSP hydrogels and *IGF2* (d), *IGF1R* (e), and *IGF1* (f) expression was investigated with qPCR on days 1, 3, 7, and 14. n=9 for each time point, from 3 independent experiments, each with 3 technical replicates. Two-way ANOVA was performed, followed by Tukey's multiple comparisons test. * indicates $p < 0.05$ between HA and RGDSP at the same time point. †, ‡, § indicates $p < 0.05$ from day 1, 3, and day 7 measurements of the same data set, respectively.

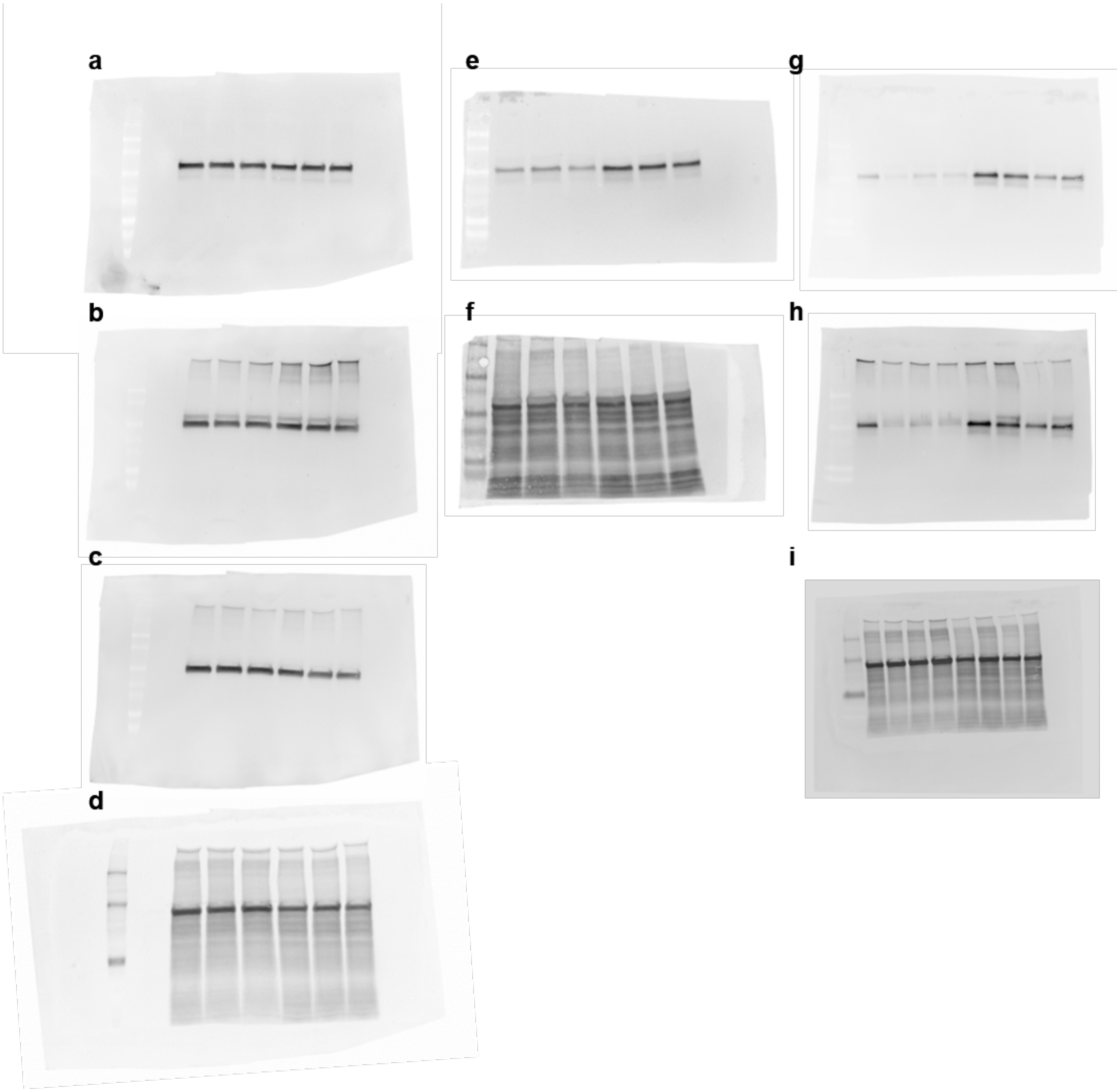

Fig. S7. (**a-d**) Original western blots for data presented in Fig.1b for keratin-5 (a), fibronectin (b), keratin-14 (c) and the total protein (d). (**e-f**) Original western blots for data presented in Fig. 1b for keratin-7 (e) and the total protein (f). (**g-i**) Original western blots for data presented in Fig. 5 for keratin-7 (g), fibronectin (h) and the total protein (i).

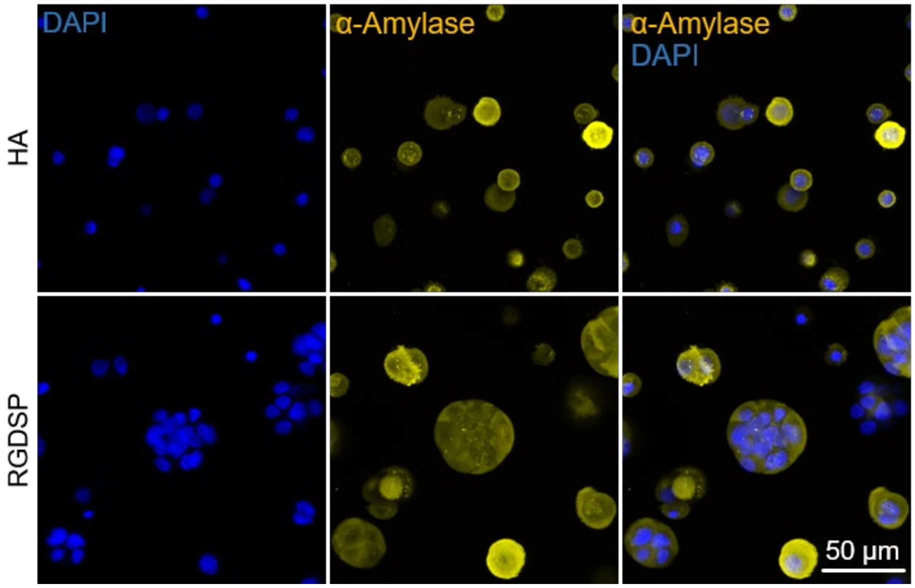

Fig. S8. hS/PCs were cultured in HA and RGDSP gels for 14 days, and ICC was conducted to visualize amylase (yellow) expression by fluorescent microscopy. Cell nuclei were counter stained in blue.

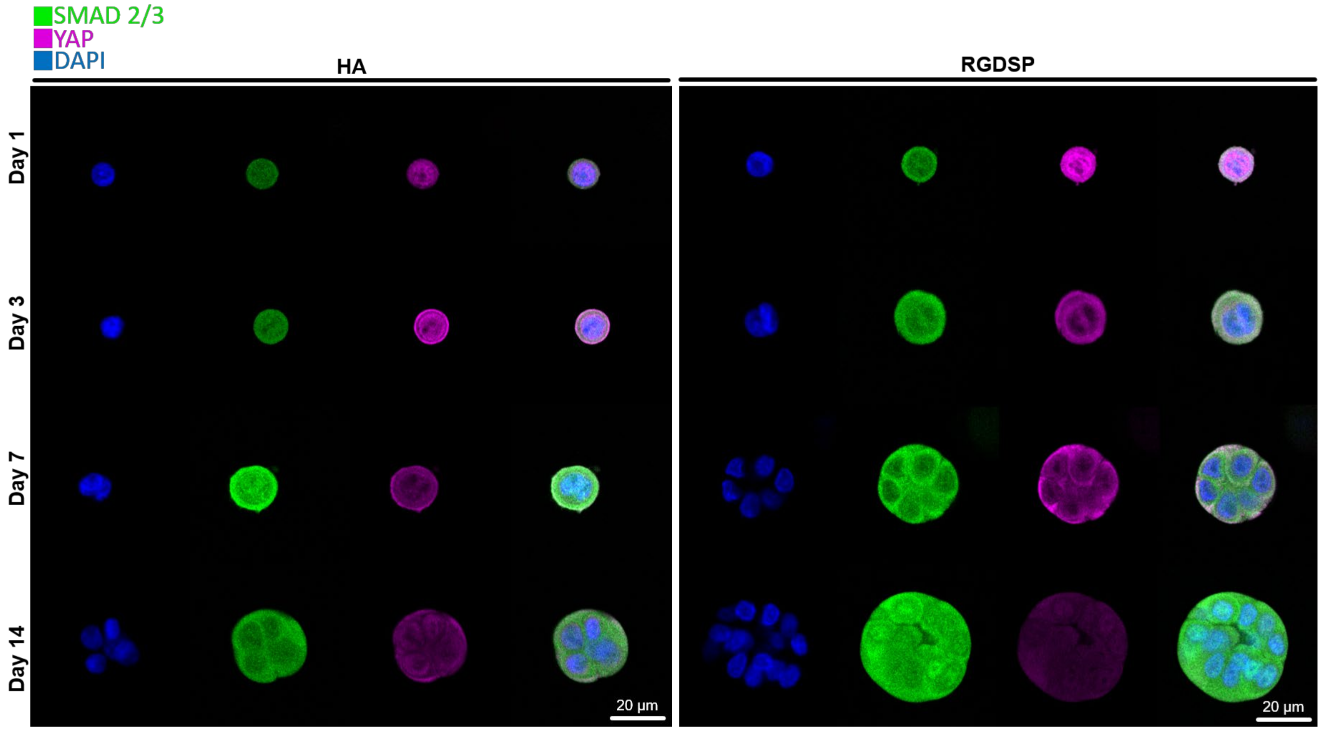

Fig. S9. Fluorescent microscopy images of hS/PCs in HA and RGDSP gels with SMAD 2/3, YAP and nuclei stained green, magenta and blue, respectively.

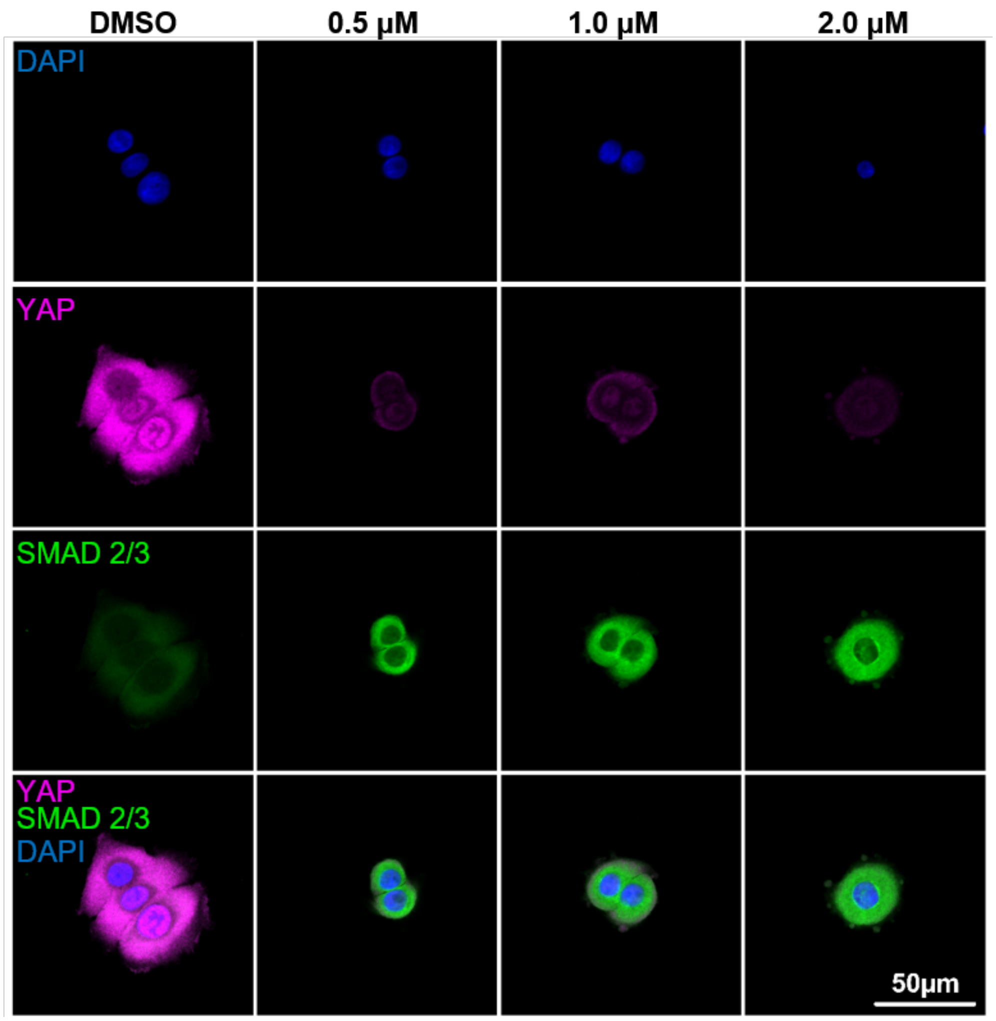

Fig. S10. hS/PCs were cultured with verteporfin (VERT, 0.5, 1.0, and 2.0 μM) or the DMSO vehicle control for 24 h before SMAD 2/3 (green) and YAP (magenta) were visualized by ICC. Nuclei were counterstained with DAPI (blue).

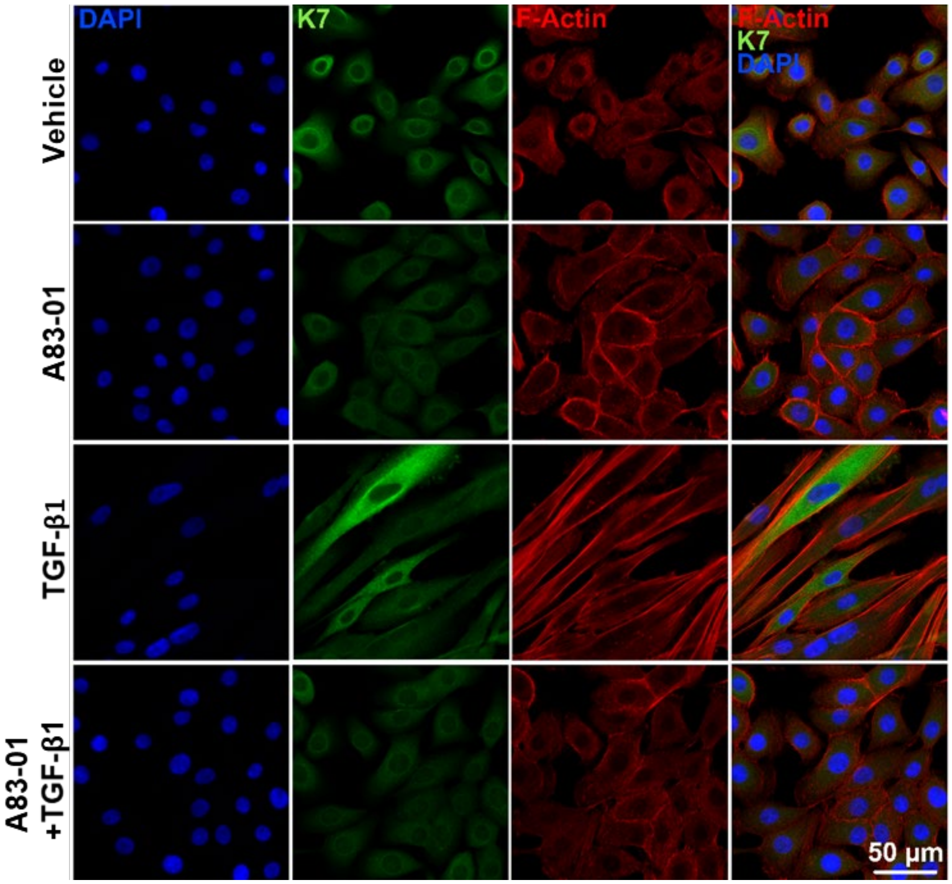

Fig. S11. hS/PCs were cultured on 2D with TGF-β1 and A83-01 for 48 h, and K7 (green), F-actin (red) and nuclei (blue) were visualized with fluorescent microscopy.

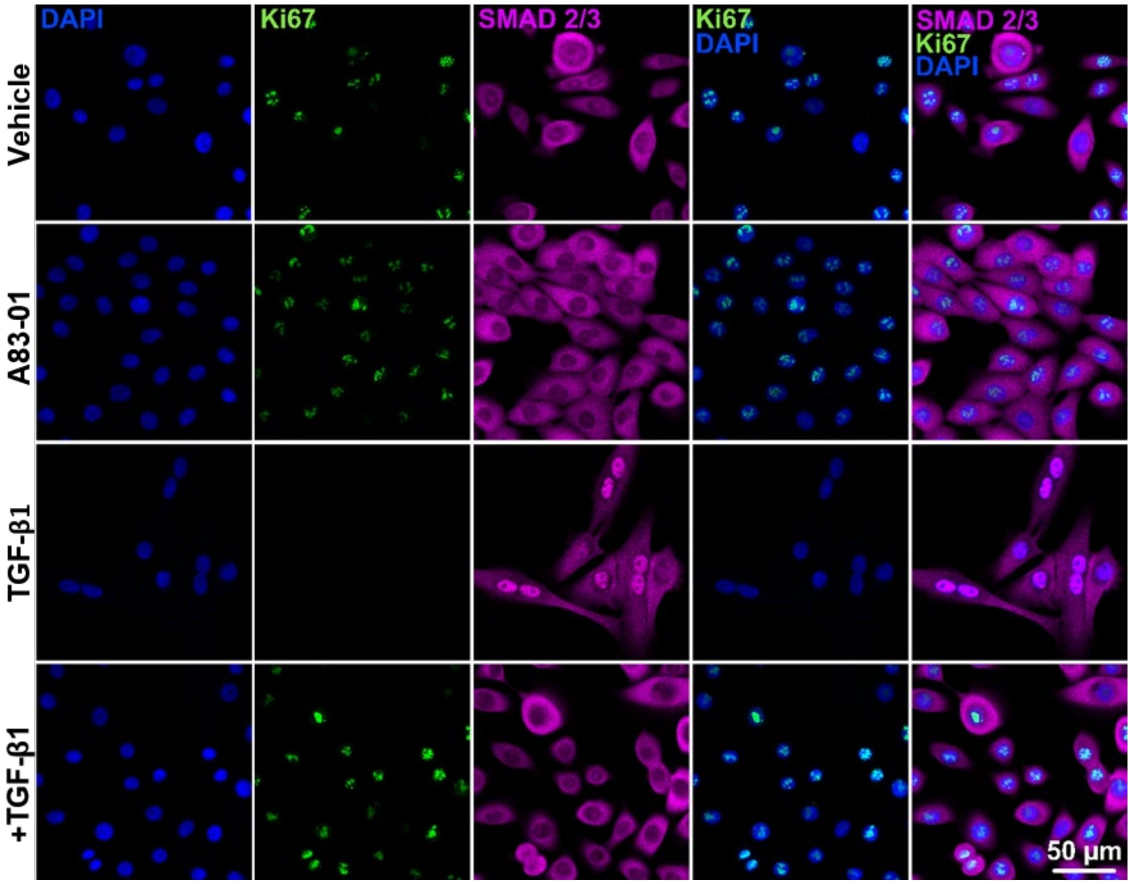

Fig S12. hS/PCs were cultured on 2D with TGF-β1 and A83-01 for 48 h, and the expressions of SMAD 2/3 (magenta) and Ki-67 (green) were investigated with ICC. Cell nuclei were counter stained by DAPI in blue.

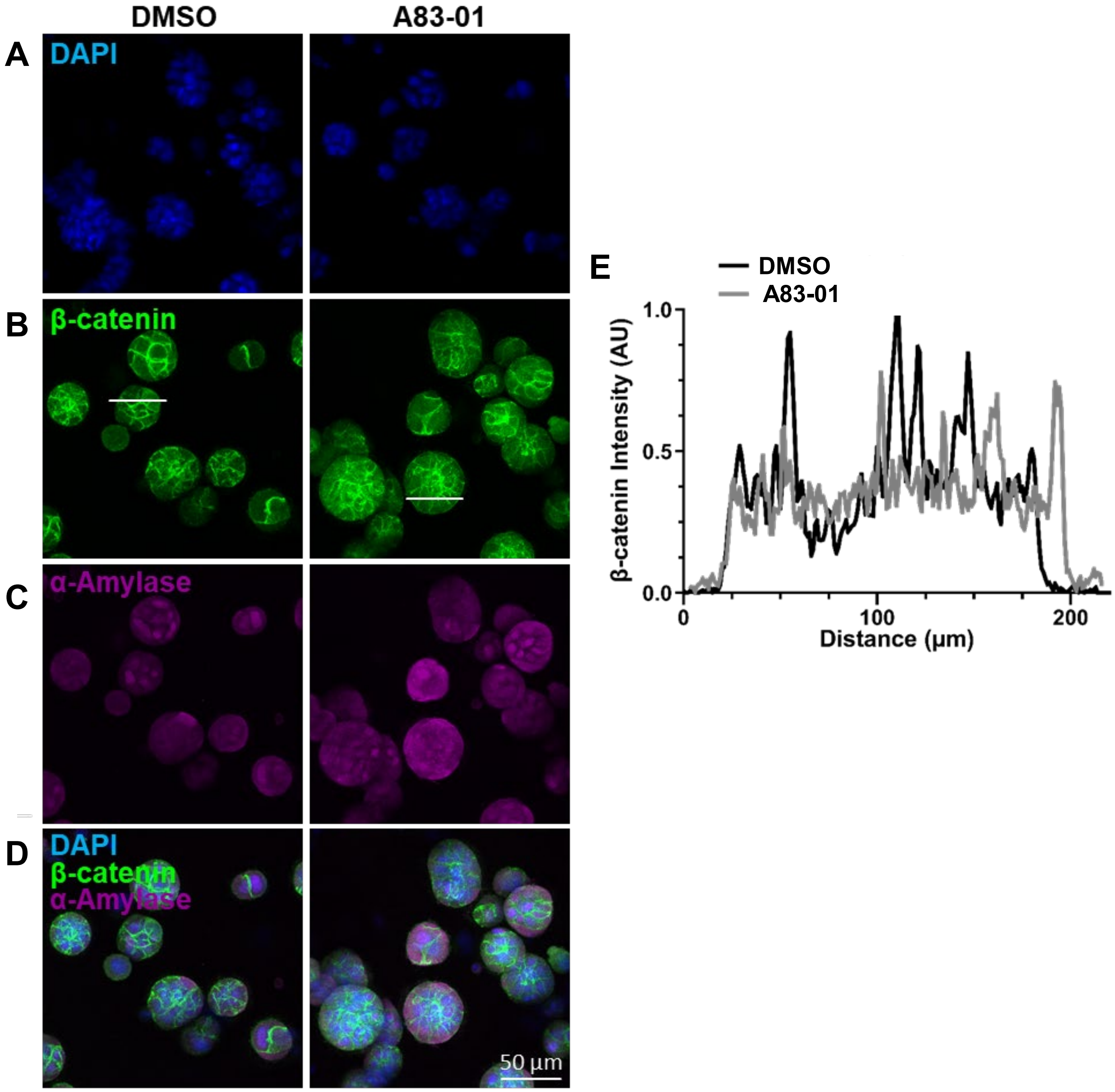

Fig S13. hS/PCs were cultured in RGDSP hydrogels for 14 days with DMSO or A83-01 and the expression of β-catenin (B) and α-amylase (C) was visualized by ICC. Nuclei were counter stained by DAPI in blue (A). The merged images are shown in D. The line profiles of β-catenin intensity shown in E correspond to the white lines shown in B.

References

1 Spandidos, A., Wang, X., Wang, H. & Seed, B. PrimerBank: a resource of human and mouse PCR primer pairs for gene expression detection and quantification. *Nucleic acids research* **38**, D792-799, doi:10.1093/nar/gkp1005 (2010).

2 Taylor, S. C. *et al.* The Ultimate qPCR Experiment: Producing Publication Quality, Reproducible Data the First Time. *Trends in biotechnology* **37**, 761-774, doi:10.1016/j.tibtech.2018.12.002 (2019).

3 Ye, J. *et al.* Primer-BLAST: a tool to design target-specific primers for polymerase chain reaction. *BMC bioinformatics* **13**, 134, doi:10.1186/1471-2105-13-134 (2012).

4 Hellemans, J., Mortier, G., De Paepe, A., Speleman, F. & Vandesompele, J. qBase relative quantification framework and software for management and automated analysis of real-time quantitative PCR data. *Genome biology* **8**, R19, doi:10.1186/gb-2007-8-2-r19 (2007).

5 Kawarada, Y. *et al.* TGF-beta induces p53/Smads complex formation in the PAI-1 promoter to activate transcription. *Scientific reports* **6**, 35483, doi:10.1038/srep35483 (2016).

6 Zhang, Y. *et al.* High throughput determination of TGFbeta1/SMAD3 targets in A549 lung epithelial cells. *PloS one* **6**, e20319, doi:10.1371/journal.pone.0020319 (2011).

7 Stein, C. *et al.* YAP1 Exerts Its Transcriptional Control via TEAD-Mediated Activation of Enhancers. *PLoS Genet* **11**, e1005465, doi:10.1371/journal.pgen.1005465 (2015).

8 Szymaniak, A. D. *et al.* The Hippo pathway effector YAP is an essential regulator of ductal progenitor patterning in the mouse submandibular gland. *Elife* **6**, doi:10.7554/eLife.23499 (2017).

9 Totaro, A. *et al.* YAP/TAZ link cell mechanics to Notch signalling to control epidermal stem cell fate. *Nature communications* **8**, 15206, doi:10.1038/ncomms15206 (2017).

10 Li, L., Hu, J. S. & Olson, E. N. Different members of the jun proto-oncogene family exhibit distinct patterns of expression in response to type beta transforming growth factor. *J Biol Chem* **265**, 1556-1562, doi:10.1016/S0021-9258(19)40053-7 (1990).

11 Hiwatashi, N. *et al.* SMAD3 expression and regulation of fibroplasia in vocal fold injury. *Laryngoscope* **127**, E308-E316, doi:10.1002/lary.26648 (2017).

12 Yeh, Y. C. *et al.* Transforming growth factor-{beta}1 induces Smad3-dependent {beta}1 integrin gene expression in epithelial-to-mesenchymal transition during chronic tubulointerstitial fibrosis. *Am J Pathol* **177**, 1743-1754, doi:10.2353/ajpath.2010.091183 (2010).

13 Cai, J. *et al.* A RhoA-YAP-c-Myc signaling axis promotes the development of polycystic kidney disease. *Genes & development* **32**, 781-793, doi:10.1101/gad.315127.118 (2018).

14 Zhang, J. *et al.* YAP-dependent induction of amphiregulin identifies a non-cell-autonomous component of the Hippo pathway. *Nat Cell Biol* **11**, 1444-1450, doi:10.1038/ncb1993 (2009).

15 Quan, C., Yan, Y., Qin, Z., Lin, Z. & Quan, T. Ezrin regulates skin fibroblast size/mechanical properties and YAP-dependent proliferation. *J Cell Commun Signal* **12**, 549-560, doi:10.1007/s12079-017-0406-6 (2018).

16 Ramdas, V., McBride, M., Denby, L. & Baker, A. H. Canonical transforming growth factor-beta signaling regulates disintegrin metalloprotease expression in experimental renal fibrosis via miR-29. *Am J Pathol* **183**, 1885-1896, doi:10.1016/j.ajpath.2013.08.027 (2013).

17 Leivonen, S. K. *et al.* TGF-beta-elicited induction of tissue inhibitor of metalloproteinases (TIMP)-3 expression in fibroblasts involves complex interplay between Smad3, p38alpha, and ERK1/2. *PloS one* **8**, e57474, doi:10.1371/journal.pone.0057474 (2013).

18 Hall, M. C. *et al.* The comparative role of activator protein 1 and Smad factors in the regulation of Timp-1 and MMP-1 gene expression by transforming growth factor-beta 1. *J Biol Chem* **278**, 10304-10313, doi:10.1074/jbc.M212334200 (2003).

19 Yuan, W. & Varga, J. Transforming growth factor-beta repression of matrix metalloproteinase-1 in dermal fibroblasts involves Smad3. *J Biol Chem* **276**, 38502-38510, doi:10.1074/jbc.M107081200 (2001).

20 Zavadil, J. *et al.* Genetic programs of epithelial cell plasticity directed by transforming growth factor-beta. *Proceedings of the National Academy of Sciences of the United States of America* **98**, 6686-6691, doi:10.1073/pnas.111614398 (2001).

21 Yang, A. T. *et al.* TGF-beta1 Induces the Dual Regulation of Hepatic Progenitor Cells with Both Anti- and Proliver Fibrosis. *Stem Cells Int* **2016**, 1492694, doi:10.1155/2016/1492694 (2016).

22 Munger, J. S. & Sheppard, D. Cross talk among TGF-beta signaling pathways, integrins, and the extracellular matrix. *Cold Spring Harbor perspectives in biology* **3**, a005017, doi:10.1101/cshperspect.a005017 (2011).

23 Wesselkamper, S. C. *et al.* Gene expression changes during the development of acute lung injury: role of transforming growth factor beta. *Am J Respir Crit Care Med* **172**, 1399-1411, doi:10.1164/rccm.200502-286OC (2005).

24 Wang, T. *et al.* YAP promotes breast cancer metastasis by repressing growth differentiation factor-15. *Biochimica et biophysica acta. Molecular basis of disease* **1864**, 1744-1753, doi:10.1016/j.bbadis.2018.02.020 (2018).
